# Cardiovascular safety of Janus kinase inhibitors in patients with rheumatoid arthritis: systematic review and network meta-analysis

**DOI:** 10.3389/fphar.2023.1237234

**Published:** 2023-08-08

**Authors:** Qige Wei, Hui Wang, Jianglin Zhao, Zhongping Luo, Chufeng Wang, Chunmei Zhu, Na Su, Shengzhao Zhang

**Affiliations:** ^1^ Department of Pharmacy, Karamay Central Hospital/Karamay Hospital of Xinjiang Uygur Autonomous Region People’s Hospital, Karamay, China; ^2^ Xinjiang Clinical Research Center for Precision Medicine of Digestive System Tumor, Karamay, China; ^3^ Xinjiang Key Laboratory of Clinical Genetic Testing and Biomedical Information, Karamay, China; ^4^ Department of Nephropathy and Rheumatology, Karamay Central Hospital/Karamay Hospital of Xinjiang Uygur Autonomous Region People’s Hospital, Karamay, China; ^5^ Department of Pharmacy, West China Hospital, Sichuan University, Chengdu, China

**Keywords:** cardiovascular safety, Janus kinase inhbitors, rheumathoid arthritis, network meta-analysis, major cardiovascular adverse events, all-cause mortality

## Abstract

**Background:** Janus kinase (JAK) inhibitors have emerged as a progressively utilized therapeutic approach for the management of rheumatoid arthritis (RA). However, the complete determination of their cardiovascular safety remains inconclusive. Hence, the primary objective of this network meta-analysis is to meticulously assess and juxtapose the cardiovascular risks linked to distinct JAK inhibitors employed in RA patients.

**Methods:** A systematic review and network meta-analysis were meticulously conducted, encompassing a collection of randomized controlled trials (RCTs) that focused on investigating the incidence of major adverse cardiovascular events (MACE) and all-cause mortality associated with Janus kinase (JAK) inhibitors administered to patients with rheumatoid arthritis (RA). Extensive exploration was performed across multiple electronic databases, incorporating studies published until March 2023. To be included in this analysis, the RCTs were required to involve adult participants diagnosed with RA who received treatment with JAK inhibitors. To ensure accuracy, two authors independently undertook the selection of eligible RCTs and meticulously extracted aggregate data. In order to examine the outcomes of MACE and all-cause mortality, a frequentist graph theoretical approach within network meta-analyses was employed, utilizing random-effects models. Third study has been registered on PROSPERO under the reference CRD42022384611.

**Findings:** A specific selection encompassing a total of 14 meticulously chosen randomized controlled trials was undertaken, wherein 13,524 patients were assigned randomly to distinct treatment interventions. The analysis revealed no notable disparity in the occurrence of major adverse cardiovascular events (MACE) between the interventions and the placebo group. However, in comparison to adalimumab, the employment of JAK inhibitors exhibited an association with higher rates of all-cause mortality [odds ratio (OR): 1.7, 95% confidence interval (CI): 1.02–2.81]. This observed increase in risk primarily stemmed from the usage of tofacitinib (OR: 1.9, 95% CI: 1.12–3.23). None of the other JAK inhibitors exhibited a statistically significant variance in all-cause mortality when compared to adalimumab.

**Interpretation:** Our study suggests that JAK inhibitors may not increase the risk of MACE in RA patients but may be associated with a higher risk of all-cause mortality compared to adalimumab, primarily due to tofacitinib use. Rheumatologists should carefully consider the cardiovascular risks when prescribing JAK inhibitors, particularly tofacitinib, for RA patients.

**Systematic Review Registration:** https://www.crd.york.ac.uk/PROSPERO/display_record.php?RecordID=384611, CRD42022384611.

## Introduction

Rheumatoid arthritis (RA) is a chronic immune-mediated disease that triggers systemic inflammation and autoantibody production, characterized by persistent synovitis (Scott et al., 2010; van Delft and Huizinga, 2020; Gravallese and Firestein, 2023). Uncontrolled active RA can lead to joint damage, disability, decreased quality of life, as well as cardiovascular and other complications (Figus et al., 2021). The global incidence of RA is relatively stable, around 0.5%–1.0%, and the disease is most typical in women and the elderly (Silman and Pearson, 2002; Finckh et al., 2022). Although the prevalence of RA varies among different countries and regions, with higher rates observed in industrialized nations; with the industrialization process and economic development of developing countries, the incidence rate of rheumatoid arthritis is expected to increase (Alamanos and Drosos, 2005; Tobón et al., 2010).

Pharmacological treatment is currently the primary approach for managing RA, including conventional synthetic disease-modifying antirheumatic drugs (DMARDs), biologic DMARDs, and targeted synthetic DMARDs such as JAK inhibitors (Smolen et al., 2016; Fraenkel et al., 2021; McLornan et al., 2021; Lauper et al., 2022). However, it should be noted that these drugs, especially JAK inhibitors, have the potential for serious adverse events, particularly cardiovascular events (CVEs), which may be related to the decreased rate of cholesterol ester breakdown caused by JAK inhibitors and the possible occurrence of major adverse cardiovascular events (MACE) (Baldini et al., 2021; Benucci et al., 2022; Winthrop and Cohen, 2022; Hoisnard et al., 2023; Molander et al., 2023). The US Food and Drug Administration has issued warnings regarding the increased risk of serious heart-related events, cancer, blood clots, and death associated with JAK inhibitors used to treat certain chronic inflammatory conditions (FDA, 2023).

In previous pairwise meta-analysis, no difference was found in cardiovascular outcomes between different JAK inhibitors (Xie et al., 2019). Compared to pairwise meta-analysis, network meta-analysis has the advantage of including indirect evidence and ranking all interventions. Therefore, the aim of our study is to conduct a network meta-analysis by incorporating additional data and research findings, in order to evaluate the impact of different JAK inhibitors on cardiovascular outcomes in patients with rheumatoid arthritis.

## Methods

Our study report follows the PRISMA (Preferred Reporting Items for Systematic Reviews and Meta-Analyses) 2020 and PRISMA Network Meta-analysis reporting standards to ensure compliance with high-quality reporting requirements and enhance the credibility of our research outcomes (Hutton et al., 2015; Page et al., 2021). We have registered this network meta-analysis on PROSPERO(CRD42022384611).

### Inclusion criteria

**Study type** We included randomized controlled trials (RCTs) and examined any interventions of interest (which will be defined in detail below). These RCTs were connected within a network that allowed comparison through shared comparators.

**Participants** Our study included research involving adults (aged over 18 years) who have a confirmed diagnosis of rheumatoid arthritis (diagnostic criteria as per the American College of Rheumatology’s “Diagnostic Criteria for Rheumatoid Arthritis” published in 1958, 1987, or 2010) (ROPES et al., 1958; Arnett et al., 1988; Aletaha et al., 2010).

**Interventions** The interventions of interest were JAK inhibitors that have been approved or are under development, including tofacitinib, filgotinib, baricitinib, upadacitinib, and decernotinib, regardless of whether they were used as monotherapy or in combination with conventional synthetic disease-modifying antirheumatic drugs (csDMARDs) (i.e., methotrexate) or steroids. Considering the variability of doses in clinical practice, we did not impose dose restrictions on csDMARDs and JAK inhibitors.

**Outcomes** The endpoint is to determine MACE and all-cause mortality. MACE was defined as a composite endpoint of myocardial infarction, cerebrovascular accident (ischemic and hemorrhagic strokes) or death from cardiovascular causes.

### Search methods

We conducted a specific search for relevant studies using three electronic databases (PubMed, EMBASE, and CENTRAL) from the beginning of their records until 10 March 2023. The search terms and strategy were based on the Cochrane guidelines (Cumpston et al., 2022). The details of search strategy could be found in Supplementary Appendix SA2 (Supplementary Table S2).

### Study selection

Two reviewers independently screened articles for inclusion, with the option to assess both the title/abstract and full text if necessary. Any discrepancies were resolved by consensus or through discussion with a third reviewer.

### Data extraction

Two reviewers worked in pairs to extract relevant data from the included studies into an Excel spreadsheet. Extracted information included patient baseline characteristics and outcome indicators of trials. Results data were independently extracted, with any discrepancies resolved through discussion with a third reviewer. For all trials, we also searched for data on clinical trial registry websites.

### Time point of outcome assessment

In parallel group trials, we used end-of-trial data to assess outcomes. However, in trials where treatment medications or doses were changed, we only considered the initial switch in treatment for outcome assessment to avoid interference and confusion between treatments.

### Risk of bias of studies

Two reviewers independently evaluated the risk of bias. The Risk of Bias Version 2 (RoB 2) (Sterne et al., 2019) tool developed by the Cochrane Collaboration was used to assess bias risk in the following five domains: random sequence generation, allocation concealment, blinding, completeness of outcome data, and selective outcome reporting. The studies were categorized as having “low,” “high,” or “some concerns” bias for each of the five domains, depending on the potential risk of bias in that domain. The bias risk for each outcome was assessed separately, considering that different domains may have varying levels of bias risk. We also evaluated the overall bias risk for each outcome.

### Publication bias

Publication bias was assessed using a funnel plot, and if the distribution of points on the funnel plot was relatively symmetrical, it was considered that publication bias did not exist. In addition to the funnel plot, Egger’s test, Begg-Mazumdar test, and Thompson-Sharp test were used to test for publication bias. If *p* > 0.05, it was considered that there was no publication bias (Sterne and Egger, 2001; Chaimani and Salanti, 2012). Funnel plots and related tests for publication bias were implemented using the “netmeta” package in R version 4.2.2 (Genovesa et al., 2016).

### Data analysis

Considering the potential heterogeneity between interventions and control measures we performed a network meta-analysis with a random-effects model using a frequentist graph theoretical approach. As all included outcome measures were categorical variables, we expressed the relative effects between two interventions as odds ratios (ORs) and their corresponding 95% confidence intervals (CIs) and presented them in league tables. The frequentist network meta-analysis was performed using the “netmeta” package in R 4.2.2 (Genovesa et al., 2016). Cochran’s Q test was used to assess heterogeneity in the network meta-analysis (Jackson et al., 2012), including both direct and indirect comparisons. Consistency was evaluated using node-splitting analysis (Dias et al., 2010; Higgins et al., 2012), comparing whether there was consistency between direct and indirect comparisons of each pair of interventions. Results of heterogeneity and consistency tests were considered good if *p* > 0.05, indicating heterogeneity and consistency among the included studies. We assessed the intransitivity of our results by comparing the distribution of potential effect-modifying factors, including baseline age, gender, and disease duration, for each direct comparison. The effectiveness and safety ranking of the interventions are obtained by calculating the P-score of each intervention in the outcome measures. The higher the corresponding P-score, the better the safety of the intervention (Rücker and Schwarzer, 2017). A comprehensive evaluation of effectiveness and safety ranking is presented by drawing a scatter plot based on the P-scores obtained by each intervention in each outcome measure (Veroniki et al., 2019).

For sensitivity analysis, we first conducted a Bayesian network meta-analysis using a random-effects model as an alternative approach. gemtc package in R was used for the Bayesian network meta-analysis (Gert van Valkenhoef and Joel Kuiper., 2021). Additionally, we performed frequency-based network meta-analyses as sensitivity analyses by excluding studies that did not report “all-cause mortality” as an outcome measure, studies with treatment or follow-up periods of less than 12 weeks, and studies categorized as having “some concerns” of bias. The robustness of the main analysis was evaluated based on the results of these sensitivity analyses.

All the statistical analyses described above were performed using R software version 4.2.2.

### Presentation of results

We used the “multinma” package in R to create and display a network plot illustrating the direct and indirect comparison relationships among different interventions. Each node in the network plot represents an intervention, with the size of the node reflecting the sample size. The links between nodes represent the existence of direct comparison relationships among these interventions. The thickness of the link represents the number of direct comparisons between two interventions, which is reflected by the number of solid lines connecting the two nodes.

League tables were used to summarize the research data, where each cell in the table represents the relative effect size between the intervention of the row and that of the column. The color of each cell represents the GRADE rating, indicating the strength of evidence for that comparison. Different colors are used to represent different GRADE ratings, blue for moderate quality evidence, yellow for low quality evidence, and red for very low quality evidence.

To evaluate the consistency of the results, we employed forest plots as graphical representations. Within the forest plot, each individual study was depicted by a small square or diamond, wherein the point estimate and corresponding confidence interval were illustrated as a line segment. This visual depiction allowed for a comprehensive assessment of the consistency across the included studies.

To effectively showcase transitivity, a combination of violin plots and box plots was employed. Initially, violin plots were utilized to present the distribution of data from the target studies. These plots effectively captured the shape of the distribution, providing insights into the spread and density of the data. Following the violin plots, box plots were utilized to display the mean values along with their corresponding confidence intervals. This presentation allowed for a concise representation of the central tendency and the variability of the data, facilitating a comprehensive understanding of transitivity across the analyzed studies.

### Quality of evidence (GRADE)

We evaluated the quality of the results in this study by applying the GRADE (Grading of Recommendations Assessment, Development, and Evaluation) approach specifically designed for network meta-analysis (Puhan et al., 2014; Brignardello-Petersen et al., 2018; Brignardello-Petersen et al., 2019). The rating process involved two independent reviewers conducting the rating, with any discrepancies resolved through discussion or consultation with a third reviewer. The rating results consist of high, moderate, low, and very low quality. First, the direct comparisons were evaluated based on factors like bias risk, heterogeneity, reporting bias, and whether the outcome measure is a final endpoint. Then, the indirect comparisons were rated based on the presence of moderate, low, or very low-quality evidence in the transitivity process. Lastly, the network meta-analysis results were graded based on the ratings of direct comparisons, indirect comparisons, consistency, and precision of the results. The quality rating of evidence from indirect comparisons could be lowered due to intransitivity.

The rating results include high, moderate, low, and very low quality. Very low quality indicates substantial uncertainty or major limitations in the available evidence, making it difficult to draw reliable conclusions. This can be caused by factors such as a high risk of bias, substantial heterogeneity, insufficient reporting of study methods or results, or the outcome measure not being a final endpoint. Low quality suggests limitations in the available evidence, but it is still possible to draw some conclusions with caution. Limitations could include a moderate risk of bias, moderate heterogeneity, or incomplete reporting of study methods or results. Moderate quality indicates that the available evidence is reasonably reliable, but there are still some limitations to consider. This could be due to a low risk of bias, low heterogeneity, or a satisfactory level of reporting of study methods and results. High quality means the available evidence is considered robust and reliable, with minimal limitations. This could be attributed to a low risk of bias, low heterogeneity, comprehensive reporting of study methods and results, and a strong level of confidence in the conclusions drawn. To quantify the proportional contribution of each direct comparison to each indirect and network comparison, a contribution matrix was constructed using the random walk approach (Davies et al., 2022).

## Results

### Search results and description of included studies

**FIGURE 1:**
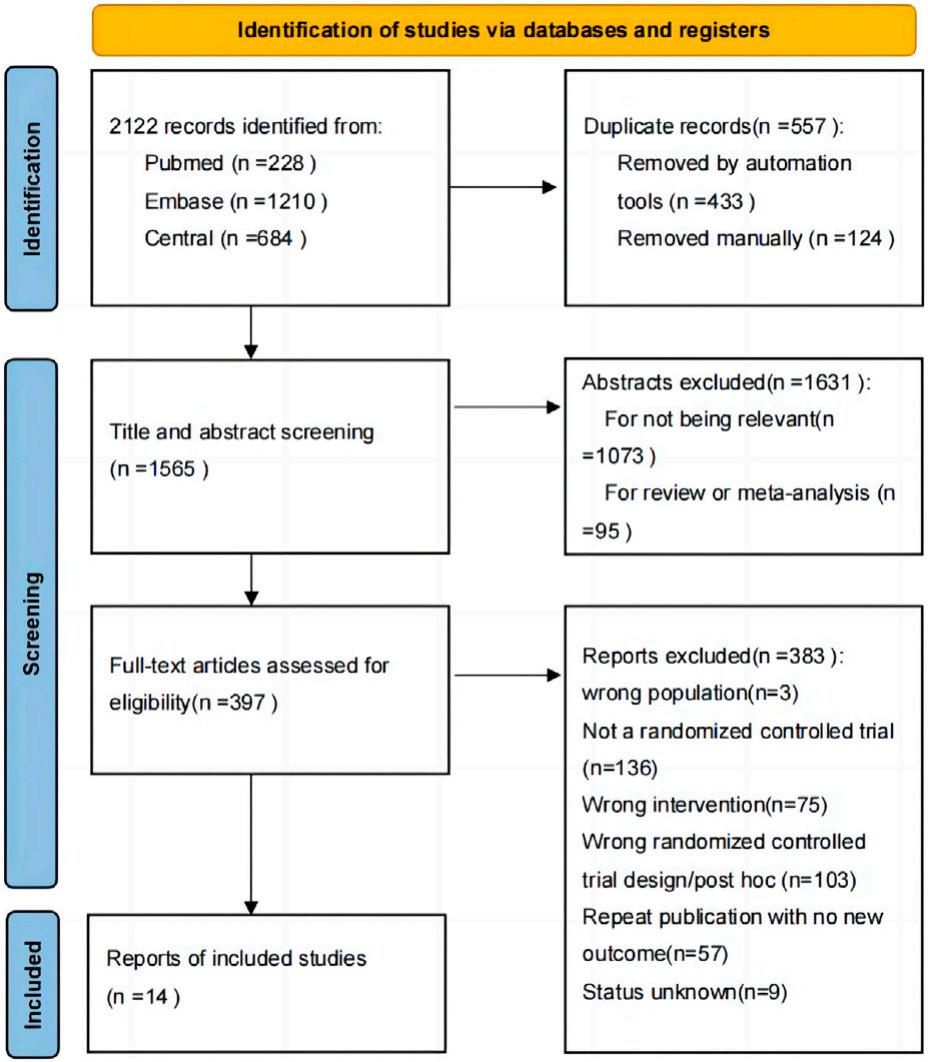
PRISMA flow diagram of the study selection process.

From 1,565 unique records, we identified 14 trials that met our inclusion criteria, involving 13,524 patients (Kremer et al., 2013; Fleischmann et al., 2015; Genovese et al., 2016; Taylor et al., 2017; Burmester et al., 2018; Genovese et al., 2018; Fleischmann et al., 2019; Smolen et al., 2019; Rubbert-Roth et al., 2020; van Vollenhoven et al., 2020; Ozdede and Yazıcı, 2022). See Figure 1 for the PRISMA flow diagram summarizing search results. The enrolled patients had an average age ranging from 50.1 to 61.4 years old, with female patients accounting for 68.00%–87.32% of the sample. The average duration of illness was between 2.6–14.5 years, and the follow-up period ranged from 4 to 52 weeks. Details of each study are presented in Supplementary Table S3.

### Results of network diagram

**FIGURE 2:**
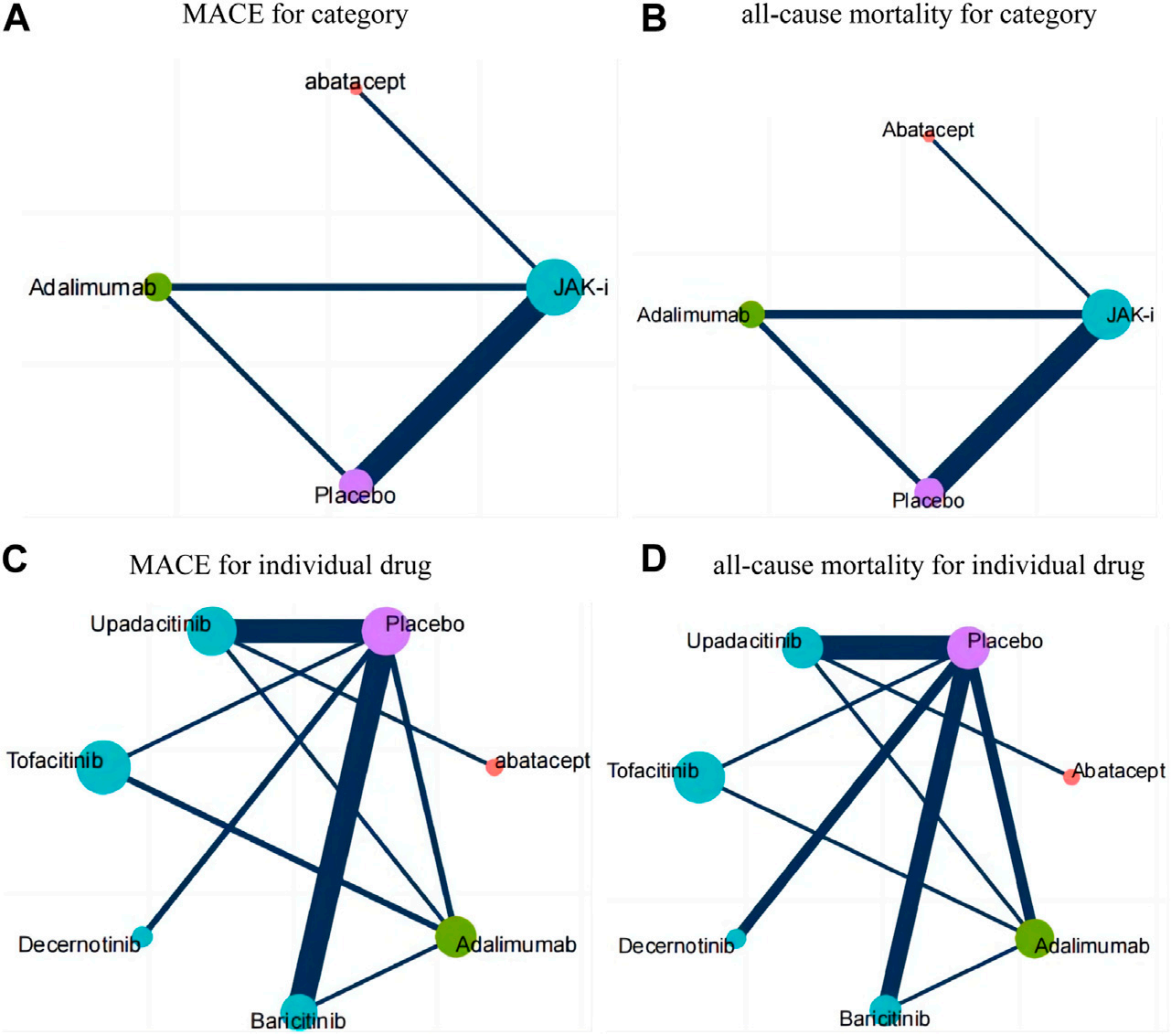
Network diagrams for each outcome measure. Annotation: Each node in the network plot represents an intervention, with the size of the node reflecting the sample size. The links between nodes represent the existence of direct comparison relationships among these interventions. The thickness of the link represents the number of direct comparisons between two interventions, which is reflected by the number of solid lines connecting the two nodes. **(A)**: MACE for category; **(B)**: all-cause mortality for category; **(C)**: MACE for individual drug; **(D)**: all-cause mortality for individual drug.

Four network diagrams were created for each of the two outcome measures, MACE and all-cause mortality, divided by category and individual drug. For individual drug, MACE was included in 14 intervention measures, while all-cause mortality was included in 7 intervention measures. By category, four intervention measures were included for both MACE and all-cause mortality. The network are shown in Figure 2.

### Risk of bias

In general, the studies included exhibit a low risk of bias. Four out of fourteen trials showed some concern in one of the five domains. One reason was lack of blinding (25%) (Ytterberg et al., 2022), another was inability to address whether missing data might lead to bias (25%) (Genovese et al., 2016), and another was absence of an independent cardiovascular adjudication committee to determine potential cardiovascular events (50%) (Fleischmann et al., 2015; Genovese et al., 2016). The specific risk of bias assessment results can be found in Supplementary Appendix SA1 Section 4. The statistical results indicate the absence of significant publication bias.

### Publication bias

The specific results of publication bias can be found in Supplementary Appendix SA1 Section 5.

### Results of network meta-analysis

**FIGURE 3:**
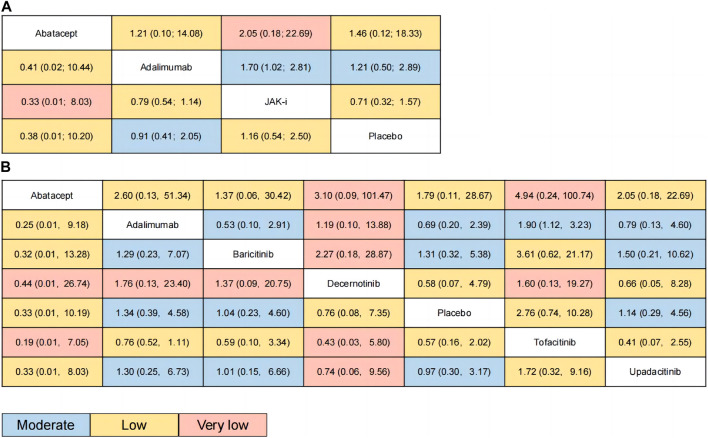
Results of network meta-analysis (relative effects league table and GRADE rating). Annotation: League tables were used to summarize the research data, where each cell in the table represents the relative effect size between the intervention of the row and that of the column. The color of each cell represents the GRADE rating, indicating the strength of evidence for that comparison. Different colors are used to represent different GRADE ratings, blue for moderate quality evidence, yellow for low quality evidence, and red for very low quality evidence. **(A)**: for category; **(B)**: for individual drug.

The relative effects of the network meta-analysis results were presented in a league table format, along with the GRADE rating results. The league table and GRADE rating results are shown in Figure 3, and details of the GRADE rating can be found in Supplementary Appendix SA1 Section 9 (Supplementary Table S5).

**FIGURE 4:**
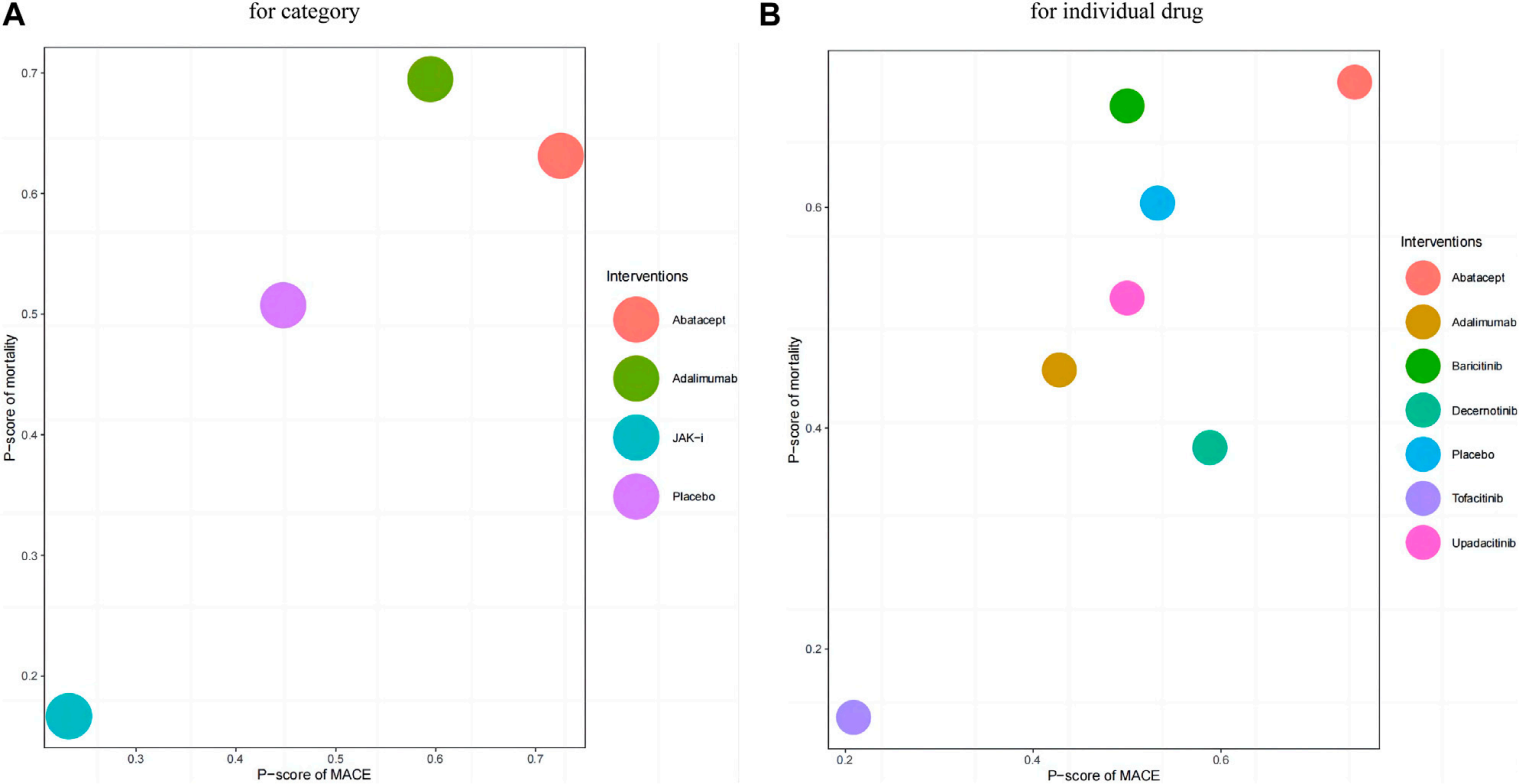
Scatter Plot of *p*-values. Annotation: In this scatter plot, the *x*-axis represents the P-score of MACE, and the *y*-axis represents the P-score of mortality. Each data point represents a study drug, and different colors indicate different study drugs. The position of each data point indicates the corresponding study’s P-score. The higher the P score, the lower the incidence of MACE and all-cause mortality for the intervention. Drugs with higher P-scores for MACE will be located on the right side of the scatter plot, while drugs with higher P-scores for mortality will be located above the scatter plot. **(A)**: for category; **(B)**: for individual drug.

Figure 3A presents the estimated effects of different categories of drugs on MACE and all-cause mortality, which are displayed in different locations. The relative effect estimates for MACE are shown in the lower left corner of the table, while the relative effect estimates for all-cause mortality are shown in the upper right corner of the table. It can be seen from the table that the differences between the different drugs in terms of MACE did not reach statistical significance, but in terms of all-cause mortality, the use of JAK inhibitors may increase overall mortality compared to adalimumab (OR: 1.7, 95% CI: 1.02–2.81). The GRADE rating, which ranges from very low to moderate, conveys information regarding the level of certainty in the findings. Based on the P-score of MACE, the sorting results are in order: Abatacept (P-score = 0.7253), Adalimumab (P-score = 0.5946), Placebo (P-score = 0.4473), JAK-i (P-score = 0.2328), respectively (see the appendix for details). For all-cause mortality, the sorting results are in order: Adalimumab (P-score = 0.6948), Abatacept (P-score = 0.6312), Placebo (P-score = 0.5073), JAK-i (P-score = 0.1667). We also presented this result using a scatter plot (as illustrated in Figure 4A).

Figure 3B presents the estimated effects of different drugs on MACE and all-cause mortality, which are displayed in different locations. The relative effect estimates for MACE are shown in the lower left corner of the table, while the relative effect estimates for all-cause mortality are shown in the upper right corner of the table. It can be seen from the table that the differences between the different drugs in terms of MACE did not reach statistical significance, but in terms of all-cause mortality, the use of Tofacitinib inhibitors may increase overall mortality compared to adalimumab (OR: 1.9, 95% CI: 1.12–3.23). The GRADE rating, which ranges from very low to moderate, conveys information regarding the level of certainty in the findings. Based on the P-score of MACE, the sorting results are in order: Abatacept (P-score = 0.7426), Decernotinib (P-score = 0.5882), Placebo (P-score = 0.5325),Baricitinib (P-score = 0.5001), Upadacitinib (P-score = 0.5001), Adalimumab (P-score = 0.4279), Tofacitinib (P-score = 0.2086). For all-cause mortality, the P-score analysis suggests that Abatacept (P-score = 0.7134), Baricitinib (P-score = 0.6919), Placebo (P-score = 0.6038), Upadacitinib (P-score = 0.5178),Adalimumab (P-score = 0.4527), Decernotinib (P-score = 0.3824),Tofacitinib (P-score = 0.1380). We also presented this result using a scatter plot (as illustrated in Figure 4B).

The results of tests for heterogeneity, consistency and transitivity in the network meta-analysis can be found in Supplementary Appendix SA1 Section 6–8. Overall, with regards to tests for heterogeneity, the network meta-analysis showed low levels of heterogeneity across all outcome measures, and the test results also showed no obvious inconsistencies or untranslatability.

The results of sensitivity analyses showed that the trend of OR values was consistent with the main analysis results, indicating good robustness of the main results of the network meta-analysis. The specific results of the sensitivity analysis can be found in Supplementary Appendix SA1 Section 10.

## Discussion

Our network meta-analysis specifically concentrated on evaluating the risk of major adverse cardiovascular events (MACE) and all-cause mortality associated with the utilization of JAK inhibitors in individuals diagnosed with rheumatoid arthritis. The findings of our analysis indicate that, in comparison to alternative treatment drugs, JAK inhibitors did not exhibit any statistically significant variances in terms of MACE occurrence or all-cause mortality, except for a noteworthy increased risk of all-cause mortality observed with Tofacitinib in comparison to Adalimumab. Our results indicate that decernotinib may have the lowest risk of major adverse cardiovascular events (MACE) among JAK inhibitors, while baricitinib had the lowest risk of all-cause mortality. However, due to the lack of randomized controlled trials (RCTs) for filgotinib that reported these outcomes, it was not included in the ranking analysis.

### Agreements and disagreements with other studies or reviews

Accumulating evidence indicates that patients diagnosed with rheumatoid arthritis (RA) face a heightened incidence of cardiovascular disease compared to the general population. This elevated risk can be attributed not only to a higher prevalence of traditional cardiovascular risk factors among RA patients but also to the presence of chronic inflammation, which serves as an independent cardiovascular risk factor. The chronic inflammatory component associated with RA contributes to the overall cardiovascular burden experienced by individuals with this condition. Hence, addressing both traditional risk factors and the underlying inflammatory processes is crucial in managing the cardiovascular health of RA patients (Van Doornum et al., 2006; Nurmohamed, 2009; Semb et al., 2020; Bridges Jr et al., 2022; Yuan et al., 2022). Hence, when making decisions regarding the choice of medication for treating rheumatoid arthritis (RA), it is imperative to take into account not only the medication’s efficacy in achieving long-term control of joint and systemic inflammation but also its potential cardiovascular risks. JAK inhibitors, being a novel class of disease-modifying antirheumatic drugs, require further evaluation in terms of their impact on cardiovascular safety. As such, a comprehensive assessment of the potential cardiovascular risks associated with JAK inhibitors is essential in order to make informed treatment decisions and ensure the overall wellbeing of RA patients (Sepriano et al., 2023).

Previous studies mainly conducted systematic review and meta-analyses of the overall adverse events of JAK inhibitors, lacking focus on the cardiovascular risk or mortality risk of JAK inhibitors for treating RA. A meta-analysis which evaluate the safety of JAK inhibitors for treating inflammatory bowel diseases or other immune-mediated diseases showed that an increased risk of herpes zoster infection among patients with immune-mediated diseases (IMID) treated with JAK inhibitors and other AEs were not increased among patients treated with JAK inhibitors (Olivera et al., 2020). Another systematic review and meta-analysis presented that any adverse events were more frequent with JAK inhibitors, and the risk for infection was higher for JAK inhibitors compared with placebo (Wang et al., 2020). Regarding cardiovascular safety, previous studies mostly discussed the relationship between JAK inhibitors and venous thromboembolism events. The results of two previous meta-analyses indicated that insufficient evidence to support the current warnings of VTE risk for JAK inhibitors in patients with IMID (Bilal et al., 2021; Yates et al., 2021). Only one previous meta-analysis evaluated impact of Janus kinase inhibitors on risk of cardiovascular events in patients with rheumatoid arthritis, the results demonstrated that The existing evidence from RCTs indicated no significant change in cardiovascular risk for Jakinib-treated patients with RA in a short-term perspective, but post-marketing data are sorely needed to ascertain their cardiovascular safety, especially at the higher dose, due to increased risk of thromboembolism events for both tofacitinib and baricitinib at higher dosage (Xie et al., 2019).

The results obtained from our network meta-analysis diverged somewhat from previous studies, and there are several potential reasons for these differences. Firstly, we included a recently published randomized controlled trial (RCT) that introduced distinct inclusion and exclusion criteria. Specifically, this RCT required that patients have at least one cardiovascular risk factor at baseline, a criterion not present in previous RCTs. Nevertheless, it is important to note that rheumatoid arthritis (RA) itself is recognized as an independent cardiovascular risk factor. Consequently, we believe that this disparity in baseline characteristics is unlikely to introduce a bias significant enough to compromise the objectivity of our conclusions. Secondly, our study focused on different outcome measures compared to previous investigations. Specifically, we specifically examined the incidence of major adverse cardiovascular events (MACE) and all-cause mortality. We conducted data analysis both by considering JAK inhibitors as a class of drugs and by analyzing individual drugs within the JAK inhibitor category. This analytical approach allowed us to more comprehensively explore whether the use of JAK inhibitors is associated with an increased risk of MACE and all-cause mortality, and to elucidate any potential disparities among specific JAK inhibitors. By considering these factors and employing a rigorous methodology, we aimed to provide a comprehensive and detailed analysis of the cardiovascular risks associated with JAK inhibitors for the treatment of RA, thereby contributing valuable insights to the existing body of knowledge.

### Strengths and weaknesses of review

Our network meta-analysis possesses several notable strengths that contribute to its significance and reliability. Firstly, to the best of our knowledge, this study represents the first network meta-analysis specifically evaluating the incidence of major adverse cardiovascular events (MACE) and all-cause mortality associated with the use of JAK inhibitors in patients diagnosed with rheumatoid arthritis (RA). By addressing this research gap, our analysis provides valuable insights into the cardiovascular risks of JAK inhibitors, filling an important knowledge void in the field. Secondly, our statistical methodology is robust and comprehensive. We ensured that all results and relevant tests, such as measures of heterogeneity, consistency, and transitivity, were properly visualized and reported. Multiple sensitivity analyses and GRADE assessments were employed to further validate the reliability and credibility of our conclusions. These rigorous analytical techniques contribute to the robustness of our findings and enhance the trustworthiness of the study. Lastly, we developed a highly precise search strategy to retrieve literature that aligns with our objectives, and meticulous methods were employed for trial selection and data extraction. By systematically screening trials and adhering to strict criteria, our aim was to gather the most relevant and reliable evidence to inform our analysis. This comprehensive approach allowed us to conduct a thorough and inclusive assessment of the existing evidence, thereby strengthening the robustness and effectiveness of our findings. Given these strengths, we believe that our network meta-analysis provides valuable and reliable insights into the cardiovascular risks associated with JAK inhibitors for the treatment of rheumatoid arthritis.

While our study offers valuable insights, it is important to acknowledge several limitations. Firstly, the number of studies included in our analysis was relatively small. However, the robustness and certainty of the evidence have been assessed using GRADE, which minimizes the impact of the limited number of studies on our research findings. Secondly, both the risks of major adverse cardiovascular events (MACE) and all-cause mortality are time-dependent variables. However, due to the limited number of studies and available data, we were unable to obtain comprehensive time-event curves or analyze time-to-event data in our study. This limitation restricts our ability to provide a detailed assessment of the time-dependent aspects of these risks. Additionally, the patients included in our study may have presented with comorbidities and were treated with different doses of the included study drugs. These factors could potentially introduce variations in baseline characteristics and treatment effects. However, we believe that our findings remain transferable and applicable, as the study population encompasses real-world patients with diverse clinical profiles. Lastly, our study did not present the absolute effects or report the relevant minimal important difference (MID) values. This decision was primarily driven by the absence of statistically significant overall relative effects and the broad confidence intervals observed in the included studies, which limited the precision of our findings. We acknowledge the importance of absolute effect analysis and MID values, and we aim to address these aspects in future research to provide a more comprehensive understanding of the clinical implications. Despite these limitations, our study contributes to the existing knowledge base on the cardiovascular risks associated with JAK inhibitors in the context of treating rheumatoid arthritis. Continued research and updates will help to further refine our understanding of these risks and provide more precise estimates in the future.

### Implications for practice

In conclusion, The analysis revealed no notable disparity in the occurrence of major adverse cardiovascular events (MACE) between the JAK inhibitors and the placebo group. However, in comparison to adalimumab, the employment of JAK inhibitors exhibited an association with higher rates of all-cause mortality. It is important to interpret our findings with caution, given the limitations of our study. However, these findings provide a foundation for further investigation in this area.

### Implications for future research

Future research should aim to validate and build upon our findings by including a larger number of studies, broadening the range of interventions examined, and exploring the underlying mechanisms that may contribute to the observed differences in all-cause mortality between JAK inhibitors and other treatment drugs. Such efforts will help to enhance our understanding of the cardiovascular risks associated with JAK inhibitors and inform clinical decision-making for the treatment of RA.

## Data Availability

The original contributions presented in the study are included in the article/Supplementary Material, further inquiries can be directed to the corresponding authors.

## References

[B1] Alamanos Y. Drosos A. A. (2005). Epidemiology of adult rheumatoid arthritis. Autoimmun. Rev. 4, 130–136. 10.1016/j.autrev.2004.09.002 15823498

[B2] Aletaha D. Neogi T. Silman A. J. Funovits J. Felson D. T. Bingham C. O. 3rd (2010). 2010 rheumatoid arthritis classification criteria: An American College of Rheumatology/European league against rheumatism collaborative initiative. Ann. Rheum. Dis. 69, 1580–1588. 10.1136/ard.2010.138461 20699241

[B3] Arnett F. C. Edworthy S. M. Bloch D. A. McShane D. J. Fries J. F. Cooper N. S. (1988). The American Rheumatism Association 1987 revised criteria for the classification of rheumatoid arthritis. Arthritis rheumatism 31, 315–324. 10.1002/art.1780310302 3358796

[B4] Baldini C. Moriconi F. R. Galimberti S. Libby P. De Caterina R. (2021). The JAK-STAT pathway: An emerging target for cardiovascular disease in rheumatoid arthritis and myeloproliferative neoplasms. Eur. Heart J. 42, 4389–4400. 10.1093/eurheartj/ehab447 34343257 PMC8572559

[B5] Benucci M. Damiani A. Infantino M. Manfredi M. Lari B. Grossi V. (2022). Cardiovascular safety, cancer and Jak-inhibitors: Differences to be highlighted. Pharmacol. Res. 183, 106359. 10.1016/j.phrs.2022.106359 35907434

[B6] Bilal J. Riaz I. B. Naqvi S. Bhattacharjee S. Obert M. R. Sadiq M. (2021). Janus kinase inhibitors and risk of venous thromboembolism: A systematic review and meta-analysis. Mayo Clin. Proc. 96, 1861–1873. 10.1016/j.mayocp.2020.12.035 33840525

[B7] Bridges S. L. Jr Niewold T. B. Merriman T. R. (2022). Is rheumatoid arthritis a causal factor in cardiovascular disease. Arthritis Rheumatol. 74, 1612–1614. 10.1002/art.42236 35583794

[B8] Brignardello-Petersen R. Bonner A. Alexander P. E. Siemieniuk R. A. Furukawa T. A. Rochwerg B. (2018). Advances in the GRADE approach to rate the certainty in estimates from a network meta-analysis. J. Clin. Epidemiol. 93, 36–44. 10.1016/j.jclinepi.2017.10.005 29051107

[B9] Brignardello-Petersen R. Mustafa R. A. Siemieniuk R. Murad M. H. Agoritsas T. Izcovich A. (2019). GRADE approach to rate the certainty from a network meta-analysis: Addressing incoherence. J. Clin. Epidemiol. 108, 77–85. 10.1016/j.jclinepi.2018.11.025 30529648

[B10] Burmester G. R. Kremer J. M. Van den Bosch F. Kivitz A. Bessette L. Li Y. (2018). Safety and efficacy of upadacitinib in patients with rheumatoid arthritis and inadequate response to conventional synthetic disease-modifying anti-rheumatic drugs (SELECT-NEXT): A randomised, double-blind, placebo-controlled phase 3 trial. Lancet 391, 2503–2512. 10.1016/S0140-6736(18)31115-2 29908669

[B11] Chaimani A. Salanti G. (2012). Using network meta-analysis to evaluate the existence of small-study effects in a network of interventions. Res. Synth. Methods 3, 161–176. 10.1002/jrsm.57 26062088

[B12] Cumpston M. S. McKenzie J. E. Welch V. A. Brennan S. E. (2022). Strengthening systematic reviews in public health: Guidance in the Cochrane handbook for systematic reviews of interventions. J. Public Health (Oxf), e588–e592. 10.1093/pubmed/fdac036 44 35352103 PMC9715291

[B13] Davies A. L. Papakonstantinou T. Nikolakopoulou A. Rücker G. Galla T. (2022). Network meta-analysis and random walks. Stat. Med. 41, 2091–2114. 10.1002/sim.9346 35293631 PMC9311228

[B14] Dias S. Welton N. J. Caldwell D. M. Ades A. E. (2010). Checking consistency in mixed treatment comparison meta-analysis. Stat. Med. 29, 932–944. 10.1002/sim.3767 20213715

[B15] Figus F. A. Piga M. Azzolin I. McConnell R. Iagnocco A. (2021). Rheumatoid arthritis: Extra-articular manifestations and comorbidities. Autoimmun. Rev. 20, 102776. 10.1016/j.autrev.2021.102776 33609792

[B16] Finckh A. Gilbert B. Hodkinson B. Bae S. C. Thomas R. Deane K. D. (2022). Global epidemiology of rheumatoid arthritis. Nat. Rev. Rheumatol. 18, 591–602. 10.1038/s41584-022-00827-y 36068354

[B17] Fleischmann R. M. Damjanov N. S. Kivitz A. J. Legedza A. Hoock T. Kinnman N. (2015). A randomized, double-blind, placebo-controlled, twelve-week, dose-ranging study of decernotinib, an oral selective JAK-3 inhibitor, as monotherapy in patients with active rheumatoid arthritis. Arthritis Rheumatol. 67, 334–343. 10.1002/art.38949 25385260

[B18] Fleischmann R. Pangan A. L. Song I. H. Mysler E. Bessette L. Peterfy C. (2019). Upadacitinib versus placebo or adalimumab in patients with rheumatoid arthritis and an inadequate response to methotrexate: Results of a phase III, double-blind, randomized controlled trial. Arthritis Rheumatol. 71, 1788–1800. 10.1002/art.41032 31287230

[B19] Fraenkel L. Bathon J. M. England B. R. St Clair E. W. Arayssi T. Carandang K. (2021). 2021 American College of Rheumatology guideline for the treatment of rheumatoid arthritis. Arthritis Rheumatol. 73, 1108–1123. 10.1002/art.41752 34101376

[B20] Genovese M. C. Fleischmann R. Combe B. Hall S. Rubbert-Roth A. Zhang Y. (2018). Safety and efficacy of upadacitinib in patients with active rheumatoid arthritis refractory to biologic disease-modifying anti-rheumatic drugs (SELECT-BEYOND): A double-blind, randomised controlled phase 3 trial. Lancet 391, 2513–2524. 10.1016/S0140-6736(18)31116-4 29908670

[B21] Genovese M. C. Kremer J. Zamani O. Ludivico C. Krogulec M. Xie L. (2016). Baricitinib in patients with refractory rheumatoid arthritis. N. Engl. J. Med. 374, 1243–1252. 10.1056/NEJMoa1507247 27028914

[B22] Genovese M. C. Smolen J. S. Weinblatt M. E. Burmester G. R. Meerwein S. Camp H. S. (2016). Efficacy and safety of ABT-494, a selective JAK-1 inhibitor, in a phase IIb study in patients with rheumatoid arthritis and an inadequate response to methotrexate. Arthritis Rheumatol. 68, 2857–2866. 10.1002/art.39808 27390150 PMC5132065

[B23] Genovese M. C. van Vollenhoven R. F. Pacheco-Tena C. Zhang Y. Kinnman N. (2016). VX-509 (decernotinib), an oral selective JAK-3 inhibitor, in combination with methotrexate in patients with rheumatoid arthritis. Arthritis Rheumatol. 68, 46–55. 10.1002/art.39473 26473751

[B24] Gravallese E. M. Firestein G. S. (2023). Rheumatoid arthritis - common origins, divergent mechanisms. N. Engl. J. Med. 388 (6), 529–542. 10.1056/NEJMra2103726 36780677

[B25] Higgins J. P. Jackson D. Barrett J. K. Lu G. Ades A. E. White I. R. (2012). Consistency and inconsistency in network meta-analysis: Concepts and models for multi-arm studies. Res. Synth. Methods 3, 98–110. 10.1002/jrsm.1044 26062084 PMC4433772

[B26] Hoisnard L. Pina Vegas L. Dray-Spira R. Weill A. Zureik M. Sbidian E. (2023). Risk of major adverse cardiovascular and venous thromboembolism events in patients with rheumatoid arthritis exposed to JAK inhibitors versus adalimumab: A nationwide cohort study. Ann. Rheum. Dis. 82, 182–188. 10.1136/ard-2022-222824 36198438

[B27] Hutton B. Salanti G. Caldwell D. M. Chaimani A. Schmid C. H. Cameron C. (2015). The PRISMA extension statement for reporting of systematic reviews incorporating network meta-analyses of health care interventions: Checklist and explanations. Ann. Intern. Med. 162, 777–784. 10.7326/M14-2385 26030634

[B28] Jackson D. White I. R. Riley R. D. (2012). Quantifying the impact of between-study heterogeneity in multivariate meta-analyses. Stat. Med. 31, 3805–3820. 10.1002/sim.5453 22763950 PMC3546377

[B29] Kremer J. Li Z. G. Hall S. Fleischmann R. Genovese M. Martin-Mola E. (2013). Tofacitinib in combination with nonbiologic disease-modifying antirheumatic drugs in patients with active rheumatoid arthritis: A randomized trial. Ann. Intern. Med. 159, 253–261. 10.7326/0003-4819-159-4-201308200-00006 24026258

[B30] Lauper K. Iudici M. Mongin D. Bergstra S. A. Choquette D. Codreanu C. (2022). Effectiveness of TNF-inhibitors, abatacept, IL6-inhibitors and JAK-inhibitors in 31 846 patients with rheumatoid arthritis in 19 registers from the 'JAK-pot' collaboration. Ann. Rheum. Dis. 81, 1358–1366. 10.1136/annrheumdis-2022-222586 35705376 PMC9484385

[B31] McLornan D. P. Pope J. E. Gotlib J. Harrison C. N. (2021). Current and future status of JAK inhibitors. Lancet 398, 803–816. 10.1016/S0140-6736(21)00438-4 34454676

[B32] Molander V. Bower H. Frisell T. Delcoigne B. Di Giuseppe D. Askling J. (2023). Venous thromboembolism with JAK inhibitors and other immune-modulatory drugs: A Swedish comparative safety study among patients with rheumatoid arthritis. Ann. Rheum. Dis. 82, 189–197. 10.1136/ard-2022-223050 36150749 PMC9887398

[B33] Nurmohamed M. T. (2009). Cardiovascular risk in rheumatoid arthritis. Autoimmun. Rev. 8, 663–667. 10.1016/j.autrev.2009.02.015 19393192

[B34] Olivera P. A. Lasa J. S. Bonovas S. Danese S. Peyrin-Biroulet L. (2020). Safety of Janus kinase inhibitors in patients with inflammatory bowel diseases or other immune-mediated diseases: A systematic review and meta-analysis. Gastroenterology 158, 1554–1573. 10.1053/j.gastro.2020.01.001 31926171

[B35] Ozdede A. Yazıcı H. (2022). Cardiovascular and cancer risk with tofacitinib in rheumatoid arthritis. N. Engl. J. Med. 386, 1766–1767. 10.1056/NEJMc2202778 35507490

[B36] Page M. J. McKenzie J. E. Bossuyt P. M. Boutron I. Hoffmann T. C. Mulrow C. D. (2021). The PRISMA 2020 statement: An updated guideline for reporting systematic reviews. BMJ 372, n71. 10.1136/bmj.n71 33782057 PMC8005924

[B37] Puhan M. A. Schünemann H. J. Murad M. H. Li T. Brignardello-Petersen R. Singh J. A. (2014). A GRADE Working Group approach for rating the quality of treatment effect estimates from network meta-analysis. BMJ 349, g5630. 10.1136/bmj.g5630 25252733

[B38] Ropes M. W. Bennett G. A. Cobb S. Jacox R. Jessar R. A. (1958). 1958 Revision of diagnostic criteria for rheumatoid arthritis. Bull. rheumatic Dis. 9, 175–176. 13596783

[B39] Rubbert-Roth A. Enejosa J. Pangan A. L. Haraoui B. Rischmueller M. Khan N. (2020). Trial of upadacitinib or abatacept in rheumatoid arthritis. N. Engl. J. Med. 383, 1511–1521. 10.1056/NEJMoa2008250 33053283

[B40] Rücker G. Schwarzer G. (2017). Resolve conflicting rankings of outcomes in network meta-analysis: Partial ordering of treatments. Res. Synth. Methods 8, 526–536. 10.1002/jrsm.1270 28982216

[B41] Scott D. L. Wolfe F. Huizinga T. W. (2010). Rheumatoid arthritis. Lancet 376 (9746), 1094–1108. 10.1016/S0140-6736(10)60826-4 20870100

[B42] Semb A. G. Ikdahl E. Wibetoe G. Crowson C. Rollefstad S. (2020). Atherosclerotic cardiovascular disease prevention in rheumatoid arthritis. Nat. Rev. Rheumatol. 16, 361–379. 10.1038/s41584-020-0428-y 32494054

[B43] Sepriano A. Kerschbaumer A. Bergstra S. A. Smolen J. S. van der Heijde D. Caporali R. (2023). Safety of synthetic and biological DMARDs: A systematic literature review informing the 2022 update of the EULAR recommendations for the management of rheumatoid arthritis. Ann. Rheum. Dis. 82, 107–118. 10.1136/ard-2022-223357 36376026

[B44] Silman A. J. Pearson J. E. (2002). Epidemiology and genetics of rheumatoid arthritis. Arthritis Res. 4 (Suppl. 3), S265–S272. 10.1186/ar578 12110146 PMC3240153

[B45] Smolen J. S. Aletaha D. McInnes I. B. (2016). Rheumatoid arthritis. Lancet 388, 2023–2038. 10.1016/S0140-6736(16)30173-8 27156434

[B46] Smolen J. S. Pangan A. L. Emery P. Rigby W. Tanaka Y. Vargas J. I. (2019). Upadacitinib as monotherapy in patients with active rheumatoid arthritis and inadequate response to methotrexate (SELECT-MONOTHERAPY): A randomised, placebo-controlled, double-blind phase 3 study. Lancet 393, 2303–2311. 10.1016/S0140-6736(19)30419-2 31130260

[B47] Sterne J. A. Egger M. (2001). Funnel plots for detecting bias in meta-analysis: Guidelines on choice of axis. J. Clin. Epidemiol. 54, 1046–1055. 10.1016/s0895-4356(01)00377-8 11576817

[B48] Sterne J. Savović J. Page M. J. Elbers R. G. Blencowe N. S. Boutron I. (2019). RoB 2: A revised tool for assessing risk of bias in randomised trials. BMJ 366, l4898. 10.1136/bmj.l4898 31462531

[B49] Taylor P. C. Keystone E. C. van der Heijde D. Weinblatt M. E. Del Carmen Morales L. Reyes Gonzaga J. (2017). Baricitinib versus placebo or adalimumab in rheumatoid arthritis. N. Engl. J. Med. 376, 652–662. 10.1056/NEJMoa1608345 28199814

[B50] Tobón G. J. Youinou P. Saraux A. (2010). The environment, geo-epidemiology, and autoimmune disease: Rheumatoid arthritis. Autoimmun. Rev. 9, A288–A292. 10.1016/j.autrev.2009.11.019 19944780

[B51] van Delft M. Huizinga T. (2020). An overview of autoantibodies in rheumatoid arthritis. J. Autoimmun. 110, 102392. 10.1016/j.jaut.2019.102392 31911013

[B52] Van Doornum S. Jennings G. L. Wicks I. P. (2006). Reducing the cardiovascular disease burden in rheumatoid arthritis. Med. J. Aust. 184, 287–290. 10.5694/j.1326-5377.2006.tb00239.x 16548834

[B53] van Vollenhoven R. Takeuchi T. Pangan A. L. Friedman A. Mohamed M. F. Chen S. (2020). Efficacy and safety of upadacitinib monotherapy in methotrexate-naive patients with moderately-to-severely active rheumatoid arthritis (SELECT-EARLY): A multicenter, multi-country, randomized, double-blind, active comparator-controlled trial. Arthritis Rheumatol. 72, 1607–1620. 10.1002/art.41384 32638504 PMC7589375

[B54] Veroniki A. A. Bender R. Glasziou P. Straus S. E. Tricco A. C. (2019). The number needed to treat in pairwise and network meta-analysis and its graphical representation. J. Clin. Epidemiol. 111, 11–22. 10.1016/j.jclinepi.2019.03.007 30905696

[B55] Wang F. Sun L. Wang S. Davis J. M. 3rd Matteson E. L. Murad M. H. (2020). Efficacy and safety of tofacitinib, baricitinib, and upadacitinib for rheumatoid arthritis: A systematic review and meta-analysis. Mayo Clin. Proc. 95, 1404–1419. 10.1016/j.mayocp.2020.01.039 32499126

[B56] Winthrop K. L. Cohen S. B. (2022). Oral surveillance and JAK inhibitor safety: The theory of relativity. Nat. Rev. Rheumatol. 18, 301–304. 10.1038/s41584-022-00767-7 35318462 PMC8939241

[B57] Xie W. Huang Y. Xiao S. Sun X. Fan Y. Zhang Z. (2019). Impact of Janus kinase inhibitors on risk of cardiovascular events in patients with rheumatoid arthritis: Systematic review and meta-analysis of randomised controlled trials. Ann. Rheum. Dis. 78, 1048–1054. 10.1136/annrheumdis-2018-214846 31088790

[B58] Yates M. Mootoo A. Adas M. Bechman K. Rampes S. Patel V. (2021). Venous thromboembolism risk with JAK inhibitors: A meta-analysis. Arthritis Rheumatol. 73, 779–788. 10.1002/art.41580 33174384

[B59] Yuan S. Carter P. Mason A. M. Yang F. Burgess S. Larsson S. C. (2022). Genetic liability to rheumatoid arthritis in relation to coronary artery disease and stroke risk. Arthritis Rheumatol. 74, 1638–1647. 10.1002/art.42239 35583917 PMC9804931

