# Supplementary material for: Cardiovascular safety of Janus kinase inhibitors in patients with rheumatoid arthritis: systematic review and network meta-analysis
Source: Front Pharmacol. 2023 Aug 8;14:1237234. doi: 10.3389/fphar.2023.1237234 (PMC10442954; doi:10.3389/fphar.2023.1237234)
Supplement: Supplementary file 1 [file DataSheet1.docx]

**Supplementary appendix**

**Supplement to:**

Cardiovascular safety of Janus kinase inhibitors in patients with rheumatoid arthritis: systematic review and network meta-analysis.

**Contents**

[Section 1. PRISMA checklist 3](#_Toc31573)

[Table S1. PRISMA checklist 3](#_Toc1517)

[Section 2. Search strategy 6](#_Toc8426)

[Table S2. Peer-reviewed literature search strategy. 6](#_Toc23574)

[Section 3. Baseline characteristics of included studies 8](#_Toc4313)

[Table S3. Baseline table 8](#_Toc24490)

[Section 4. Risk of bias assessment 11](#_Toc3096)

[Figure S1. assessment for MACE 11](#_Toc23452)

[Figure S2. Assessment for all-cause mortality 12](#_Toc20570)

[Section 5. Assessment of publication bias 13](#_Toc25394)

[5.1 By category 13](#_Toc18085)

[5.1.1 MACE 13](#_Toc15054)

[5.1.2 All-cause mortality 13](#_Toc27515)

[5.2 By individual drug 14](#_Toc14314)

[5.2.1 MACE 14](#_Toc25962)

[5.2.2 All-cause mortality 14](#_Toc23024)

[Section 6. Assessment of heterogeneity 15](#_Toc9472)

[Table S4. Assessment of heterogeneity 15](#_Toc10966)

[Section 7. Consistency assessment results 17](#_Toc373)

[7.1 By category 17](#_Toc11689)

[7.1.1 MACE 17](#_Toc8070)

[7.1.2 All-cause mortality 17](#_Toc4721)

[7.2 By individual drug 18](#_Toc7260)

[7.2.1 MACE 18](#_Toc25095)

[7.2.2 All-cause mortality 19](#_Toc1137)

[Section 8. Transitivity assessment 21](#_Toc10716)

[8.1 By category 21](#_Toc7889)

[8.1.1 Age 21](#_Toc11972)

[8.1.2 Duration disease 22](#_Toc5927)

[8.1.3 Gender 22](#_Toc10043)

[8.2 By individual drug 23](#_Toc9591)

[8.2.1 Age 23](#_Toc16584)

[8.1.2 Duration disease 24](#_Toc2172)

[8.1.3 Gender 24](#_Toc4445)

[Section 9. Results of GRADE assessment 25](#_Toc4084)

[Table S5. Results of GRADE assessment 25](#_Toc23790)

[Section 10. The results of sensitivity analyses 28](#_Toc7788)

[Table S6. Results of sensitivity analyses 28](#_Toc6299)

# Section 1. PRISMA checklist

**Table S1.** **PRISMA checklist**

| **Section and Topic** | **Item #** | **Checklist item** | **Location where item is reported** |
| --- | --- | --- | --- |
| **TITLE** | | |  |
| Title | 1 | Identify the report as a systematic review. | Lines 1-3 |
| **ABSTRACT** | | |  |
| Abstract | 2 | See the PRISMA 2020 for Abstracts checklist. | Lines 13-41 |
| **INTRODUCTION** | | |  |
| Rationale | 3 | Describe the rationale for the review in the context of existing knowledge. | Lines 44-57 |
| Objectives | 4 | Provide an explicit statement of the objective(s) or question(s) the review addresses. | Lines 58-60 |
| **METHODS** | | |  |
| Eligibility criteria | 5 | Specify the inclusion and exclusion criteria for the review and how studies were grouped for the syntheses. | Lines 86-101 |
| Information sources | 6 | Specify all databases, registers, websites, organisations, reference lists and other sources searched or consulted to identify studies. Specify the date when each source was last searched or consulted. | Lines 103-107 |
| Search strategy | 7 | Present the full search strategies for all databases, registers and websites, including any filters and limits used. | Section 2 in supplementary materials |
| Selection process | 8 | Specify the methods used to decide whether a study met the inclusion criteria of the review, including how many reviewers screened each record and each report retrieved, whether they worked independently, and if applicable, details of automation tools used in the process. | Lines 109-112 |
| Data collection process | 9 | Specify the methods used to collect data from reports, including how many reviewers collected data from each report, whether they worked independently, any processes for obtaining or confirming data from study investigators, and if applicable, details of automation tools used in the process. | Lines 114-119 |
| Data items | 10a | List and define all outcomes for which data were sought. Specify whether all results that were compatible with each outcome domain in each study were sought (e.g. for all measures, time points, analyses), and if not, the methods used to decide which results to collect. | Lines 114-119 |
|  | 10b | List and define all other variables for which data were sought (e.g. participant and intervention characteristics, funding sources). Describe any assumptions made about any missing or unclear information. | Lines121-124 |
| Study risk of bias assessment | 11 | Specify the methods used to assess risk of bias in the included studies, including details of the tool(s) used, how many reviewers assessed each study and whether they worked independently, and if applicable, details of automation tools used in the process. | Lines 126-133 |
| Effect measures | 12 | Specify for each outcome the effect measure(s) (e.g. risk ratio, mean difference) used in the synthesis or presentation of results. | Lines 143-148 |
| Synthesis methods | 13a | Describe the processes used to decide which studies were eligible for each synthesis (e.g. tabulating the study intervention characteristics and comparing against the planned groups for each synthesis (item #5)). | Lines 148-152 |
|  | 13b | Describe any methods required to prepare the data for presentation or synthesis, such as handling of missing summary statistics, or data conversions. | Lines 121-124 |
|  | 13c | Describe any methods used to tabulate or visually display results of individual studies and syntheses. | Lines 152-153 |
|  | 13d | Describe any methods used to synthesize results and provide a rationale for the choice(s). If meta-analysis was performed, describe the model(s), method(s) to identify the presence and extent of statistical heterogeneity, and software package(s) used. | Lines 142-168 |
|  | 13e | Describe any methods used to explore possible causes of heterogeneity among study results (e.g. subgroup analysis, meta-regression). | Lines 148-153 |
|  | 13f | Describe any sensitivity analyses conducted to assess robustness of the synthesized results. | Lines 161-167 |
| Reporting bias assessment | 14 | Describe any methods used to assess risk of bias due to missing results in a synthesis (arising from reporting biases). | Lines 131-133 |
| Certainty assessment | 15 | Describe any methods used to assess certainty (or confidence) in the body of evidence for an outcome. | Lines 145-148 |
| **RESULTS** | | |  |
| Study selection | 16a | Describe the results of the search and selection process, from the number of records identified in the search to the number of studies included in the review, ideally using a flow diagram. | Figure 1, lines223-227 |
|  | 16b | Cite studies that might appear to meet the inclusion criteria, but which were excluded, and explain why they were excluded. | Figure 1 |
| Study characteristics | 17 | Cite each included study and present its characteristics. | Section 3 in supplementary materials  lines 185-188 |
| Risk of bias in studies | 18 | Present assessments of risk of bias for each included study. | Section 4 in supplementary materials  Lines227-230 |
| Results of individual studies | 19 | For all outcomes, present, for each study: (a) summary statistics for each group (where appropriate) and (b) an effect estimate and its precision (e.g. confidence/credible interval), ideally using structured tables or plots. | Figure 2,3, 4 |
| Results of syntheses | 20a | For each synthesis, briefly summarise the characteristics and risk of bias among contributing studies. | lines 239-246 |
|  | 20b | Present results of all statistical syntheses conducted. If meta-analysis was done, present for each the summary estimate and its precision (e.g. confidence/credible interval) and measures of statistical heterogeneity. If comparing groups, describe the direction of the effect. | Figure 2, 3, 4; lines172-176 |
|  | 20c | Present results of all investigations of possible causes of heterogeneity among study results. | Section 6-8 in supplementary materials  Lines182-185 |
|  | 20d | Present results of all sensitivity analyses conducted to assess the robustness of the synthesized results. | Section 10 in supplementary materials  lines 287-289 |
| Reporting biases | 21 | Present assessments of risk of bias due to missing results (arising from reporting biases) for each synthesis assessed. | Section 5 in supplementary materials  lines 194-219 |
| Certainty of evidence | 22 | Present assessments of certainty (or confidence) in the body of evidence for each outcome assessed. | Section 9 in supplementary materials |
| **DISCUSSION** | | |  |
| Discussion | 23a | Provide a general interpretation of the results in the context of other evidence. | Lines 293-301 |
|  | 23b | Discuss any limitations of the evidence included in the review. | Lines 374-385 |
|  | 23c | Discuss any limitations of the review processes used. | Lines 385-391 |
|  | 23d | Discuss implications of the results for practice, policy, and future research. | Lines 403-408 |
| **OTHER INFORMATION** | | |  |
| Registration and protocol | 24a | Provide registration information for the review, including register name and registration number, or state that the review was not registered. | Lines 80-84 |
|  | 24b | Indicate where the review protocol can be accessed, or state that a protocol was not prepared. |  |
|  | 24c | Describe and explain any amendments to information provided at registration or in the protocol. |  |
| Support | 25 | Describe sources of financial or non-financial support for the review, and the role of the funders or sponsors in the review. |  |
| Competing interests | 26 | Declare any competing interests of review authors. |  |
| Availability of data, code and other materials | 27 | Report which of the following are publicly available and where they can be found: template data collection forms; data extracted from included studies; data used for all analyses; analytic code; any other materials used in the review. |  |

# Section 2. Search strategy

**Table S2. Peer-reviewed literature search strategy.**

From date of database inception to 10 March 2023

| Data base | Search history |
| --- | --- |
| Pubmed | "arthritis, rheumatoid"[MeSH Terms] AND ("Janus Kinase Inhibitors"[MeSH Terms] OR "Janus Kinase Inhibitors"[Pharmacological Action] OR "baricitinib"[Supplementary Concept] OR "2 2 1h pyrrolo 2 3 b pyridin 3 yl pyrimidin 4 yl amino 2 methyl n 2 2 2 trifluoroethyl butanamide"[Supplementary Concept] OR "GLPG0634"[Supplementary Concept] OR ("tofacitinib"[Supplementary Concept] OR "tofacitinib"[All Fields] OR "tofacitinib s"[All Fields]) OR "tofacitinib"[Supplementary Concept] OR "upadacitinib"[Supplementary Concept]) AND ("Randomized Controlled Trial"[Publication Type] OR "Randomized Controlled Trials as Topic"[MeSH Terms] OR "randomized controlled trial, veterinary"[Publication Type] OR "Controlled Clinical Trial"[Publication Type]) |
| EMbase | #1 rheumatoid arthritis.mp. [mp=title, abstract, heading word, drug trade name, original title, device manufacturer, drug manufacturer, device trade name, keyword heading word, floating subheading word, candidate term word]  #2 janus kinase inhibitor.mp. [mp=title, abstract, heading word, drug trade name, original title, device manufacturer, drug manufacturer, device trade name, keyword heading word, floating subheading word, candidate term word]  #3 baricitinib.mp. [mp=title, abstract, heading word, drug trade name, original title, device manufacturer, drug manufacturer, device trade name, keyword heading word, floating subheading word, candidate term word]  #4 decernotinib.mp. [mp=title, abstract, heading word, drug trade name, original title, device manufacturer, drug manufacturer, device trade name, keyword heading word, floating subheading word, candidate term word]  #5 filgotinib.mp. [mp=title, abstract, heading word, drug trade name, original title, device manufacturer, drug manufacturer, device trade name, keyword heading word, floating subheading word, candidate term word]  #6 tofacitinib.mp. [mp=title, abstract, heading word, drug trade name, original title, device manufacturer, drug manufacturer, device trade name, keyword heading word, floating subheading word, candidate term word]  #7 upadacitinib.mp. [mp=title, abstract, heading word, drug trade name, original title, device manufacturer, drug manufacturer, device trade name, keyword heading word, floating subheading word, candidate term word]  #8 2 or 3 or 4 or 5 or 6 or 7  #9 randomized controlled trial.mp. [mp=title, abstract, heading word, drug trade name, original title, device manufacturer, drug manufacturer, device trade name, keyword heading word, floating subheading word, candidate term word]  #10 1 and 8 and 9 |
| CENTRAL | #1 rheumatoid arthritis.mp. [mp=title, original title, abstract, floating sub-heading word, mesh headings, heading words, keyword]  #2 janus kinase inhibitor.mp. [mp=title, original title, abstract, floating sub-heading word, mesh headings, heading words, keyword]  #3 baricitinib.mp. [mp=title, original title, abstract, floating sub-heading word, mesh headings, heading words, keyword]  #4 decernotinib.mp. [mp=title, original title, abstract, floating sub-heading word, mesh headings, heading words, keyword]  #5 filgotinib.mp. [mp=title, original title, abstract, floating sub-heading word, mesh headings, heading words, keyword]  #6 tofacitinib.mp. [mp=title, original title, abstract, floating sub-heading word, mesh headings, heading words, keyword]  #7 upadacitinib.mp. [mp=title, original title, abstract, floating sub-heading word, mesh headings, heading words, keyword]  #8 2 or 3 or 4 or 5 or 6 or 7  #9 randomized controlled trial.mp. [mp=title, original title, abstract, floating sub-heading word, mesh headings, heading words, keyword]  #10 1 and 8 and 9 |

# Section 3. Baseline characteristics of included studies

**Table S3. Baseline table**

| Study | Register number | Interventions | Sample size | Age | % of female | Disease duration | Outcome | Follow up |
| --- | --- | --- | --- | --- | --- | --- | --- | --- |
| Joel Kremer2013 | NCT00856544 | Placebo | 79 | 50.8±11.2 | 79.75 | 9.5 | ①② | 52w |
|  |  | Placebo | 80 | 53.3±10.8 | 75.00 | 10.2 |  |  |
|  |  | Tofacitinib, 5 mg bid | 315 | 52.7±11.7 | 83.81 | 8.1 |  |  |
|  |  | Tofacitinib, 10mg bid | 318 | 51.9±11.8 | 81.13 | 9.2 |  |  |
| Roy M. Fleischmann2015 | NCT01052194 | Placebo | 41 | 54.9 ± 10.6 | 78.05 | 10.0±9.6 | ①② | 12w |
|  |  | Decernotinib_25mg bid | 41 | 56.8± 9.5 | 78.05 | 8.5±7.7 |  |  |
|  |  | Decernotinib_50mg bid | 41 | 55.6±11.3 | 82.93 | 6.3± 5.5 |  |  |
|  |  | Decernotinib_100mg bid | 40 | 56.5± 8.9 | 85.00 | 6.7±6.2 |  |  |
|  |  | Decernotinib_150mg bid | 41 | 57.0± 9.3 | 82.93 | 7.1±7.6 |  |  |
| Mark C. Genovese2016a | NCT02066389 | Placebo | 50 | 55±12 | 76.00 | 5.9±5.3 | ① | 12w |
|  |  | Upadacitinib_3mg bid | 50 | 53±12 | 80.00 | 3.9±3.8 |  |  |
|  |  | Upadacitinib_6mg bid | 50 | 55±12 | 68.00 | 7.0±5.5 |  |  |
|  |  | Upadacitinib_12mg bid | 50 | 56±12 | 82.00 | 9.3±8.6 |  |  |
|  |  | Upadacitinib_18mg bid | 50 | 55±14 | 84.00 | 7.3±7.9 |  |  |
|  |  | Upadacitinib_24mg qd | 49 | 56±12 | 85.71 | 8.3±7.1 |  |  |
| Mark C. Genovese2016b | NCT2011-004419-22 | Placebo | 71 | 52.7±131.2 | 87.32 | 7.2±7.5 | ①② | 24w |
|  |  | Decernotinib_100mg qd | 71 | 53.5±11.3 | 71.83 | 6.5±6.2 |  |  |
|  |  | Decernotinib_150mg qd | 72 | 50.1±11.8 | 80.56 | 8.1±8.8 |  |  |
|  |  | Decernotinib_200mg qd | 72 | 53.2±13.2 | 77.78 | 7.2±6.5 |  |  |
|  |  | Decernotinib_100mg bid | 72 | 55.7±12.2 | 86.11 | 7.7±7.5 |  |  |
| Maxime Dougados2016 | NCT01721057 | Placebo | 228 | 51±13 | 82.89 | 7±8 | ①② | 24w |
|  |  | Baricitinib_2mg qd | 229 | 52±12 | 80.35 | 8±8 |  |  |
|  |  | Baricitinib_4mg qd | 227 | 52±12 | 82.38 | 8±8 |  |  |
| Mark C2016 | NCT01721044 | Placebo | 176 | 56±11 | 82.39 | 14±10 | ①② | 24w |
|  |  | Baricitinib_2mg qd | 174 | 55±11 | 78.74 | 14±8 |  |  |
|  |  | Baricitinib_4mg qd | 177 | 56±11 | 84.18 | 14±9 |  |  |
| Peter C2017 | NCT01710358 | Placebo | 488 | 53±2 | 78.28 | 10±9 | ①② | 52w |
|  |  | Baricitinib_4mg qd | 487 | 54±2 | 77.00 | 10±9 |  |  |
|  |  | Adalimumab_40mg qow | 330 | 53±12 | 76.06 | 10±9 |  |  |
| Mark C Genovese2018 | NCT02706847 | Placebo | 169 | 57.6±11.4 | 84.62 | 14.5±9.2 | ①② | 12w |
|  |  | Upadacitinib_15mg qd | 164 | 56.3(11.3) | 83.54 | 12.4±9.4 |  |  |
|  |  | Upadacitinib_30mg qd | 165 | 57.3±11.6 | 83.64 | 12.7±9.7 |  |  |
| Gerd R Burmester2018 | NCT02675426 | Placebo | 221 | 56.0±12.2 | 75.11 | 7.2±7.5 | ① | 12w |
|  |  | Upadacitinib_15mg qd | 221 | 55.3±11.5 | 82.35 | 7.3±7.9 |  |  |
|  |  | Upadacitinib_30mg qd | 219 | 55.8±11.3 | 78.54 | 7.3±7.9 |  |  |
| Josef S Smolen2019 | NCT02706951 | Placebo | 216 | 55.3±11.1 | 82.87 | 5.8±6.6 | ①② | 14w |
|  |  | Upadacitinib_15mg qd | 217 | 54.5±12.2 | 80.18 | 7.5±8.9 |  |  |
|  |  | Upadacitinib_30mg qd | 215 | 53.1±12.7 | 79.07 | 6.5±7.0 |  |  |
| Roy Fleischmann MD2019 | NCT02629159 | Placebo | 651 | 54±12 | 78.65 | 8±8 | ①② | 48w |
|  |  | Upadacitinib_15mg qd | 651 | 54±12 | 80.03 | 8±8 |  |  |
|  |  | Adalimumab_40mg qow | 327 | 54±12 | 79.20 | 8±8 |  |  |
| Andrea Rubbert-Roth2020 | NCT03086343 | Upadacitinib_15mg qd | 303 | 55.3±11.4 | 82.18 | 12.4±9.5 | ①② | 24w |
|  |  | Abatacept_ intravenous | 309 | 55.8±11.9 | 81.88 | 11.8±8.3 |  |  |
| Ronald van Vollenhoven2020 | NCT02706873 | Placebo | 314 | 53.3±12.9 | 76.43 | 2.6±5.1 | ①② | 24w |
|  |  | Upadacitinib_15mg qd | 317 | 51.9±12.6 | 76.03 | 2.9±5.4 |  |  |
|  |  | Upadacitinib_30mg qd | 314 | 54.9±12.6 | 76.43 | 2.8±5.6 |  |  |
| Steven R. 2022 | NCT02092467 | Adalimumab_40mg qow | 1451 | 60.8±6.8 | 76.98 |  | ①② | 63w |
|  |  | Tofacitinib, 5 mg bid | 1455 | 61.4±7.1 | 80.34 |  |  |  |
|  |  | Tofacitinib, 10mg bid | 1456 | 61.3±7.5 | 77.20 |  |  |  |

Notes: ①: Major Adverse Cardiovascular Events (MACE); ②: All-cause mortality; qd=once daily, bid=Twice Daily, qow=every other week.

# Section 4. Risk of bias assessment

**Figure S1. assessment for MACE**

| Author | Study ID | D1 | D2 | D3 | D4 | D5 | Overall |
| --- | --- | --- | --- | --- | --- | --- | --- |
| Josef S Smolen2019 | NCT02706951 | **Low** | **Low** | **Low** | **Low** | **Low** | **Low** |
| Mark C Genovese2018 | NCT02706847 | **Low** | **Low** | **Low** | **Low** | **Low** | **Low** |
| Gerd R Burmester2018 | NCT02675426 | **Low** | **Low** | **Low** | **Low** | **Low** | **Low** |
| Steven R. 2022 | NCT02092467 | **Low** | **Some concerns** | **Low** | **Low** | **Low** | **Some concerns** |
| Mark C2016 | NCT01721044 | **Low** | **Low** | **Low** | **Low** | **Low** | **Low** |
| Peter C2017 | NCT01710358 | **Low** | **Low** | **Low** | **Low** | **Low** | **Low** |
| Maxime Dougados2016 | NCT01721057 | **Low** | **Low** | **Low** | **Low** | **Low** | **Low** |
| Roy Fleischmann MD2019 | NCT02629159 | **Low** | **Low** | **Low** | **Low** | **Low** | **Low** |
| Andrea Rubbert-Roth2020 | NCT03086343 | **Low** | **Low** | **Low** | **Low** | **Low** | **Low** |
| Ronald van Vollenhoven2020 | NCT02706873 | **Low** | **Low** | **Low** | **Low** | **Low** | **Low** |
| Joel Kremer2013 | NCT00856544 | **Low** | **Low** | **Some concerns** | **Low** | **Low** | **Some concerns** |
| Mark C. Genovese2016a | NCT02066389 | **Low** | **Low** | **Low** | **Low** | **Low** | **Low** |
| Mark C. Genovese2016b | NCT2011-004419-22 | **Low** | **Low** | **Low** | **Some concerns** | **Low** | **Some concerns** |
| Roy M. Fleischmann2015 | NCT01052194 | **Low** | **Low** | **Low** | **Some concerns** | **Low** | **Some concerns** |
| D1 Randomisation process D2 Deviations from the intended interventions D3 Missing outcome data D4 Measurement of the outcome D5 Selection of the reported result | | | | | | | |

**Figure S2. Assessment for all-cause mortality**

| Author | Study ID | D1 | D2 | D3 | D4 | D5 | Overall |
| --- | --- | --- | --- | --- | --- | --- | --- |
| Josef S Smolen2019 | NCT02706951 | **Low** | **Low** | **Low** | **Low** | **Low** | **Low** |
| Mark C Genovese2018 | NCT02706847 | **Low** | **Low** | **Low** | **Low** | **Low** | **Low** |
| Steven R. 2022 | NCT02092467 | **Low** | **Some concerns** | **Low** | **Low** | **Low** | **Some concerns** |
| Mark C2016 | NCT01721044 | **Low** | **Low** | **Low** | **Low** | **Low** | **Low** |
| Peter C2017 | NCT01710358 | **Low** | **Low** | **Low** | **Low** | **Low** | **Low** |
| Maxime Dougados2016 | NCT01721057 | **Low** | **Low** | **Low** | **Low** | **Low** | **Low** |
| Roy Fleischmann MD2019 | NCT02629159 | **Low** | **Low** | **Low** | **Low** | **Low** | **Low** |
| Andrea Rubbert-Roth2020 | NCT03086343 | **Low** | **Low** | **Low** | **Low** | **Low** | **Low** |
| Ronald van Vollenhoven2020 | NCT02706873 | **Low** | **Low** | **Low** | **Low** | **Low** | **Low** |
| Joel Kremer2013 | NCT00856544 | **Low** | **Low** | **Some concerns** | **Low** | **Low** | **Some concerns** |
| Mark C. Genovese2016b | NCT2011-004419-22 | **Low** | **Low** | **Low** | **Some concerns** | **Low** | **Some concerns** |
| Roy M. Fleischmann2015 | NCT01052194 | **Low** | **Low** | **Low** | **Some concerns** | **Low** | **Some concerns** |
| D1 Randomisation process D2 Deviations from the intended interventions D3 Missing outcome data D4 Measurement of the outcome D5 Selection of the reported result | | | | | | | |

# Section 5. Assessment of publication bias

**5.1 By category**

5.1.1 MACE


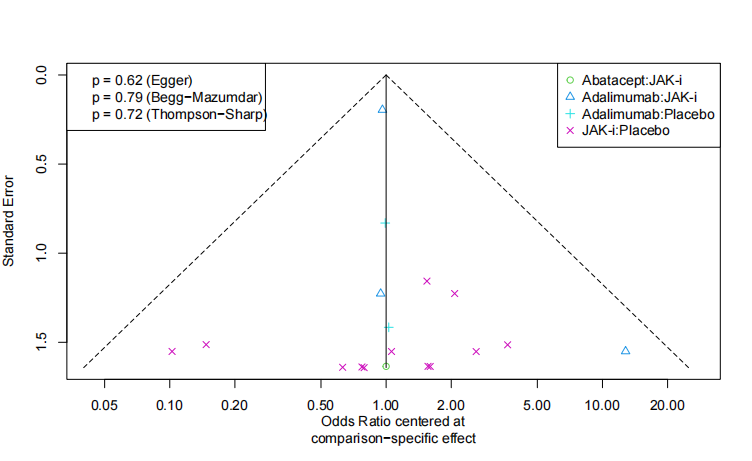


5.1.2 All-cause mortality


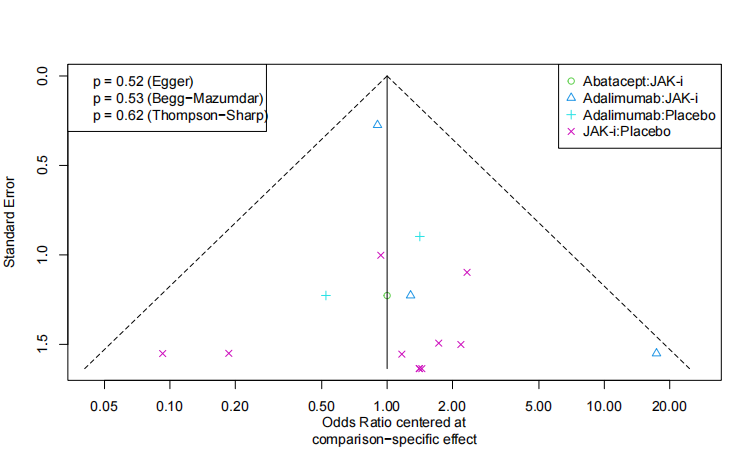


**5.2 By individual drug**

5.2.1 MACE


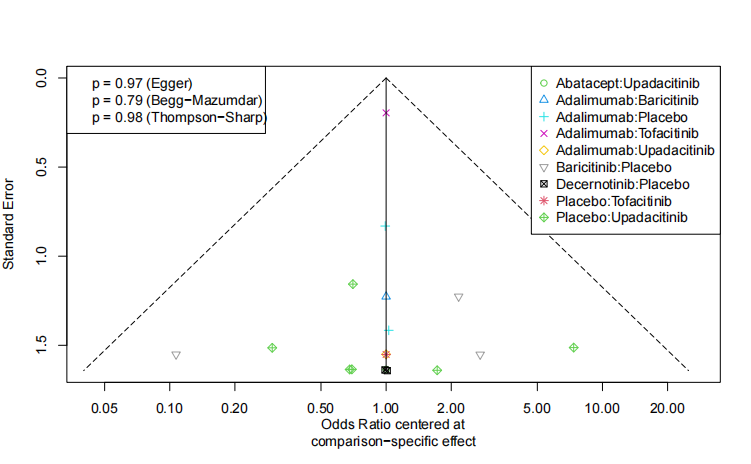


5.2.2 All-cause mortality


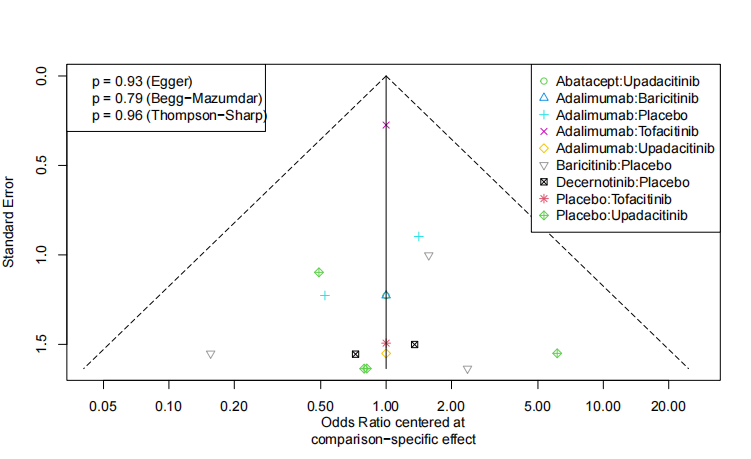


# Section 6. Assessment of heterogeneity

**Table S4. Assessment of heterogeneity**

| **By category** | | | | |
| --- | --- | --- | --- | --- |
| **Outcome measure** | **Type of comparison and compared intervention measures** | **Q statistic** | **Degrees of freedom (df)** | **P-value** |
| **MACE** | **Design-specific decomposition of within-designs Q statistic** | | | |
|  | Placebo:JAK-i | 3.66 | 9 | 0.9322 |
|  | Placebo:Adalimumab:JAK-i | 2.17 | 2 | 0.3375 |
|  | **Between-designs Q statistic after detaching of single designs** | | | |
|  | Placebo:Adalimumab:JAK-i | 0.04 | 1 | 0.8334 |
|  | Placebo:JAK-i | 0.07 | 1 | 0.4027 |
|  | Placebo:Adalimumab:JAK-i | 0 | 0 | - |
|  | **Q statistic to assess consistency under the assumption ofa full design-by-treatment interaction random effects model**：Q= o.82 , df=2, P-value=0.6626, τ=0 , τ2=0 | | | |
| **All-cause mortality** | **Design-specific decomposition of within-designs Q statistic** | | | |
|  | Placebo:JAK-i | 3.34 | 7 | 0.8516 |
|  | Placebo:Adalimumab:JAK-i | 1.74 | 2 | 0.4189 |
|  | **Between-designs Q statistic after detaching of single designs** | | | |
|  | Adalimumab:JAK-i | 0.36 | 1 | 0.5471 |
|  | Placebo:JAK-i | 2.12 | 1 | 0.1451 |
|  | Placebo:Adalimumab:JAK-i | 0 | 0 | - |
|  | **Q statistic to assess consistency under the assumption ofa full design-by-treatment interaction random effects model**：Q= 2.16 , df=2, P-value=0.3394, τ=0 , τ2=0 | | | |
| **By individual drug** | | | | |
| **MACE** | **Design-specific decomposition of within-designs Q statistic** | | | |
|  | Placebo:Baricitinib | 2.18 | 1 | 0.1402 |
|  | Placebo:Decernotinib | 0 | 1 | 0.9921 |
|  | Placebo:Upadacitinib | 0.62 | 4 | 0.9608 |
|  | **Between-designs Q statistic after detaching of single designs** | | | |
|  | Adalimumab:Tofacitinib | 3.02 | 3 | 0.3893 |
|  | Placebo:Baricitinib | 2.31 | 3 | 0.51 |
|  | Placebo:Tofacitinib | 3.02 | 3 | 0.3893 |
|  | Placebo:Upadacitinib | 0.89 | 3 | 0.8284 |
|  | Placebo:Adalimumab:Baricitinib | 2.31 | 2 | 0.3146 |
|  | Placebo:Adalimumab:Upadacitinib | 0.72 | 2 | 0.6983 |
|  | **Q statistic to assess consistency under the assumption ofa full design-by-treatment interaction random effects model**：Q= 3.16 , df=4, P-value=0.5309, τ=0 , τ2=0 | | | |
| **All-cause mortality** | **Design-specific decomposition of within-designs Q statistic** | | | |
|  | Placebo:Baricitinib | 1.46 | 1 | 0.2271 |
|  | Placebo:Decernotinib | 0.08 | 1 | 0.7721 |
|  | Placebo:Upadacitinib | 0.1 | 2 | 0.9532 |
|  | **Between-designs Q statistic after detaching of single designs** | | | |
|  | Adalimumab:Tofacitinib | 3.27 | 3 | 0.3513 |
|  | Placebo:Baricitinib | 2.61 | 3 | 0.4564 |
|  | Placebo:Tofacitinib | 3.27 | 3 | 0.3513 |
|  | Placebo:Upadacitinib | 1.17 | 3 | 0.7606 |
|  | Placebo:Adalimumab:Baricitinib | 2.08 | 2 | 0.3534 |
|  | Placebo:Adalimumab:Upadacitinib | 0.53 | 2 | 0.7690 |
|  | **Q statistic to assess consistency under the assumption ofa full design-by-treatment interaction random effects model**：Q= 3.36 , df=4, P-value=0.4989, τ=0 , τ2=0 | | | |

# Section 7. Consistency assessment results

**7.1 By category**

7.1.1 MACE


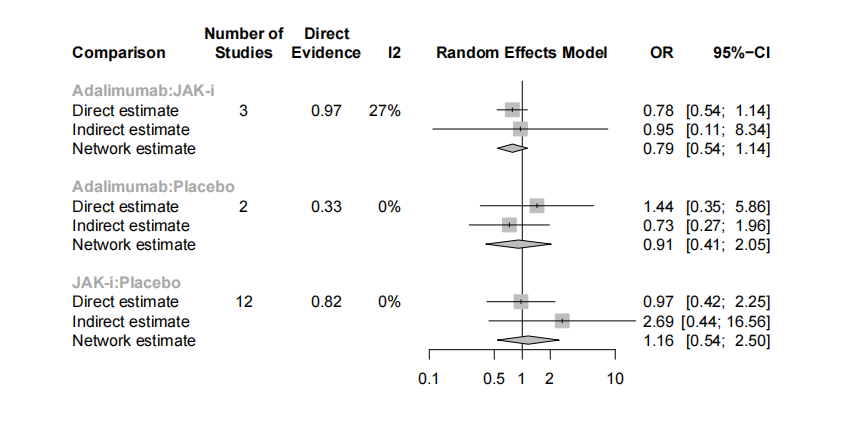


7.1.2 All-cause mortality


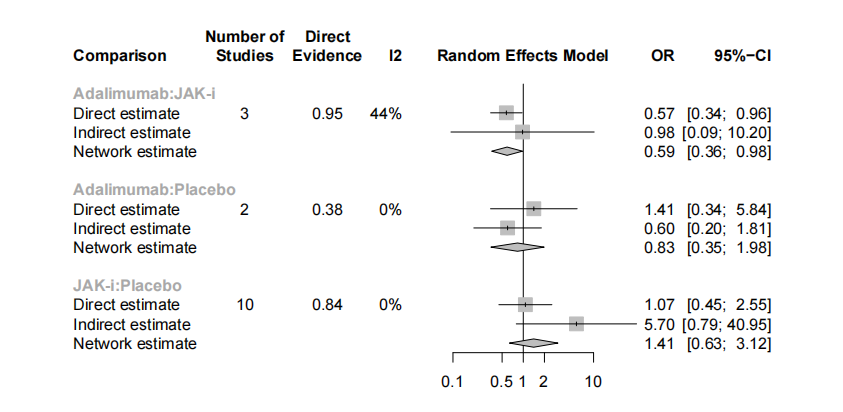


**7.2 By individual drug**

7.2.1 MACE


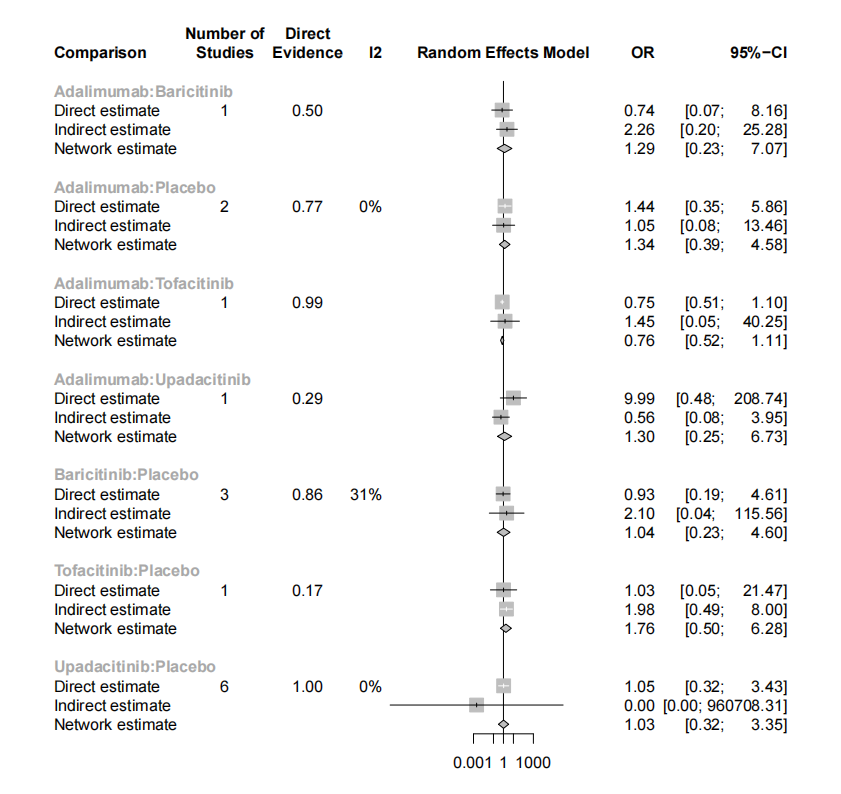


7.2.2 All-cause mortality


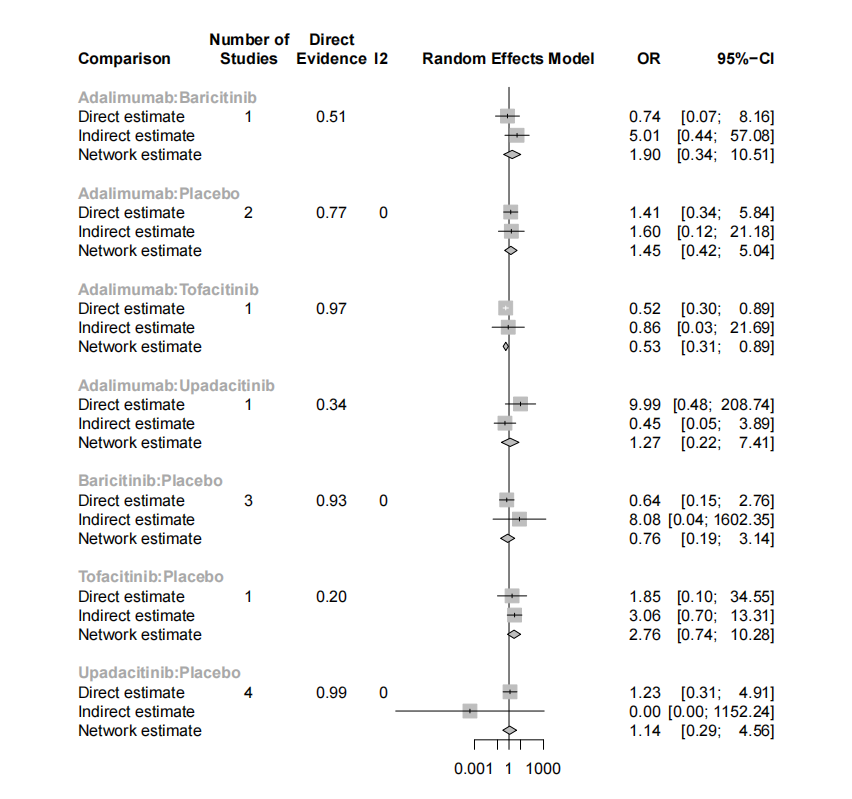


# Section 8. Transitivity assessment

**8.1 By category**

8.1.1 Age


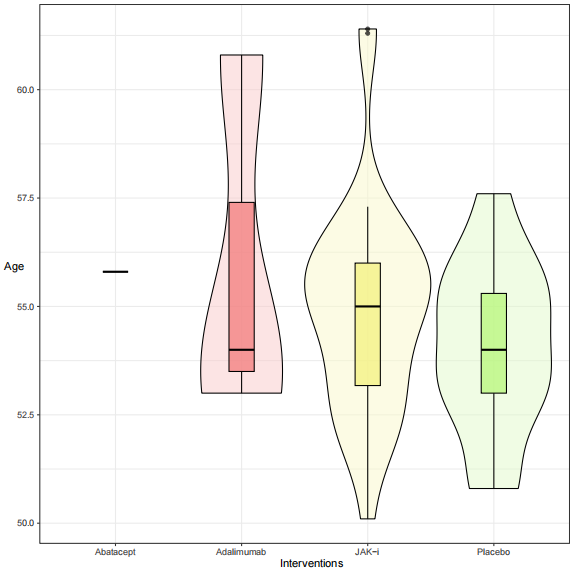


8.1.2 Duration disease


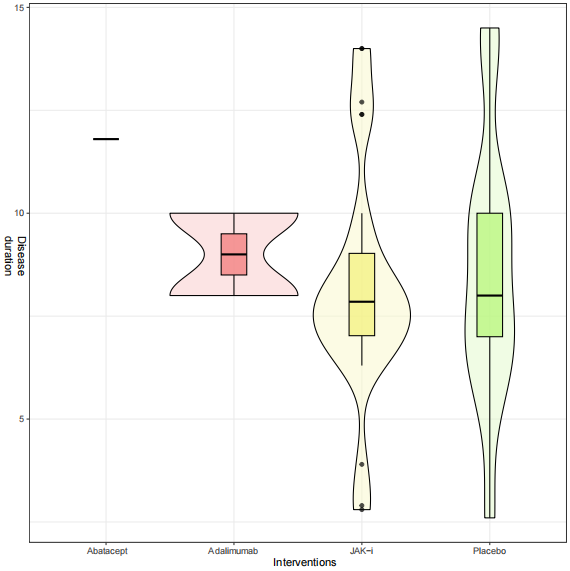


8.1.3 Gender


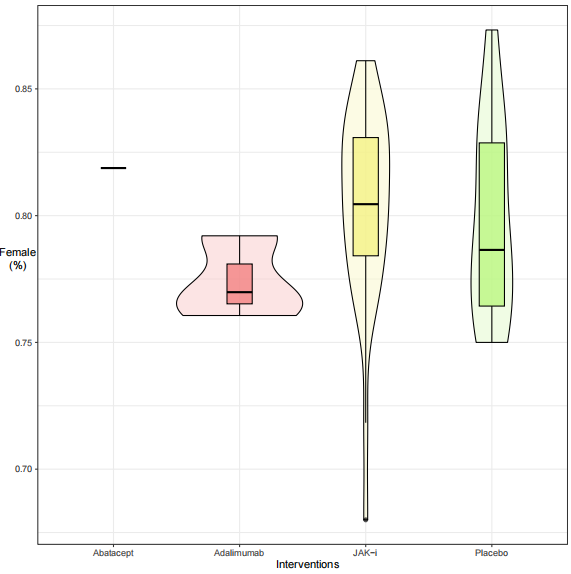


**8.2 By individual drug**

8.2.1 Age


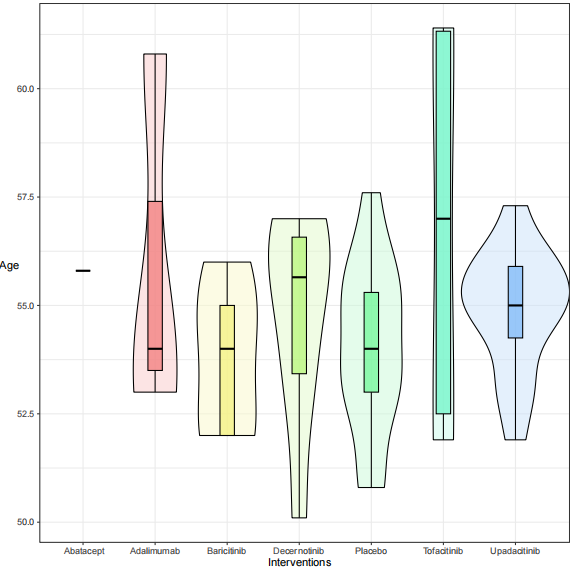


8.1.2 Duration disease


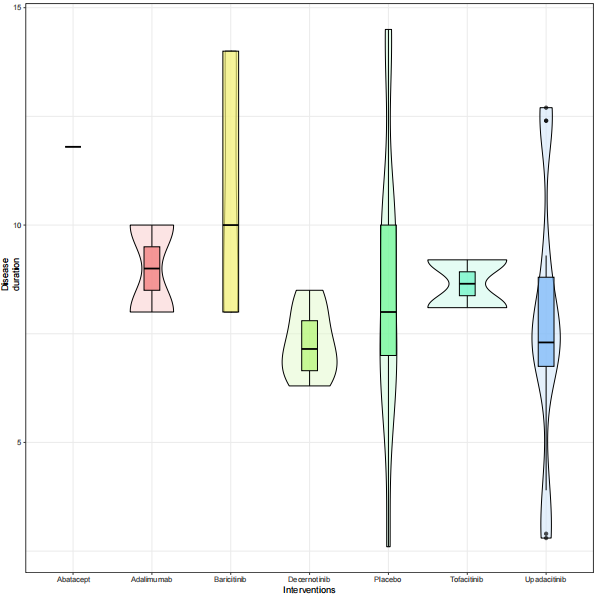


8.1.3 Gender


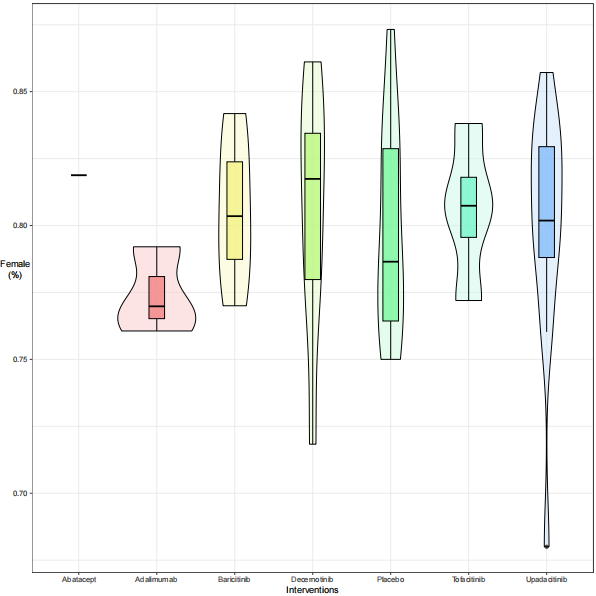


# Section 9. Results of GRADE assessment

**Table S5. Results of GRADE assessment**

| By category | | | | | | | |
| --- | --- | --- | --- | --- | --- | --- | --- |
|  | Comparative intervention measures | Direct comparison results | GRADE rating | Indirect comparison results | GRADE rating | Network meta-analysis results | GRADE rating |
| All-cause mortality | Abatacept vs Adalimumab | - | - | 0.83(0.07,9.69) | High | 0.83(0.07,9.69) | Low^Ⅳ^ |
|  | Abatacept vs JAK-i | - | - | 0.49(0.04,5.42) | Moderate^Ⅱ^ | 0.49(0.04,5.42) | Very low^Ⅳ^ |
|  | Abatacept vs Placebo | - | - | 0.69(0.05,8.66) | High | 0.69(0.05,8.66) | Low^Ⅳ^ |
|  | Adalimumab vs JAK-i | 0.57（0.34，0.96） | Moderate^Ⅰ^ | 0.98（0.09，10.2） | Moderate^Ⅱ^ | 0.59（0.36，0.98） | Moderate |
|  | Adalimumab vs placebo | 1.41（0.34，5.84） | High | 0.60（0.20，1.81） | High | 0.83（0.35，1.98） | Moderate^Ⅲ^ |
|  | JAK-i vs Placebo | 1.07（0.45，2.55） | Moderate^Ⅰ^ | 5.70（0.79，40.95） | Moderate^Ⅱ^ | 1.41(0.63,3.12) | Low^Ⅲ^ |
| MACE | Abatacept vs Adalimumab | - | - | 1.41(0.63,3.12) | High | 0.41(0.02,10.44) | Low^Ⅳ^ |
|  | Abatacept vs JAK-i | - | - | 0.33(0.01,8.03) | Moderate^Ⅱ^ | 0.33(0.01,8.03) | Very low^Ⅳ^ |
|  | Abatacept vs placebo | - | - | 0.38(0.01,10.2) | High | 0.38(0.01,10.2) | Low^Ⅳ^ |
|  | Adalimumab vs JAK-i | 0.78（0.54，1.14） | Moderate^Ⅰ^ | 0.95（0.11，8.34） | Moderate^Ⅱ^ | 0.79(0.54,1.14) | Low^Ⅲ^ |
|  | Adalimumab vs placebo | 1.44（0.35，5.86） | High | 0.73（0.27，1.96） | High | 0.91(0.41,2.05) | Moderate^Ⅲ^ |
|  | JAK-i vs placebo | 0.97（0.42） | Moderate^Ⅰ^ | 2.69（0.44，16.56） | Moderate^Ⅱ^ | 1.16（0.54，2.50） | Low^Ⅲ^ |
| By individual drug | | | | | | | |
| All-cause mortality | Abatacept vs Adalimumab | - | - | 0.38(0.02,7.6) | High | 0.38(0.02,7.6) | Low^Ⅳ^ |
|  | Abatacept vs Baricitinib | - | - | 0.73(0.03,16.28) | High | 0.73(0.03,16.28) | Low^Ⅳ^ |
|  | Abatacept vs Decernotinib | - | - | 0.32(0.01,10.58) | Moderate^Ⅱ^ | 0.32(0.01,10.58) | Very low^Ⅳ^ |
|  | Abatacept vs Placebo | - | - | 0.56(0.03,8.96) | High | 0.56(0.03,8.96) | Low^Ⅳ^ |
|  | Abatacept vs Tofacitinib | - | - | 0.20(0.01,4.13) | Moderate^Ⅱ^ | 0.20(0.01,4.13) | Very low^Ⅳ^ |
|  | Abatacept vs Upadacitinib | 0.49(0.04,5.42) | High | - |  | 0.49(0.04,5.42) | Low^Ⅳ^ |
|  | Adalimumab vs Baricitinib | 0.74(0.07,8.16) | High | 5.01(0.44,57.08) | High | 1.90(0.34,10.51) | Moderate^Ⅲ^ |
|  | Adalimumab vs Decernotinib | - | - | 0.84(0.07,9.78) | Moderate^Ⅱ^ | 0.84(0.07,9.78) | Very low^Ⅳ^ |
|  | Adalimumab vs Placebo | 1.41(0.34,5.84) | High | 1.60(0.12,21.18) | High | 1.45 (0.42， 5.04) | Moderate^Ⅲ^ |
|  | Adalimumab vs Tofacitinib | 0.52(0.30,0.89) | Moderate^Ⅰ^ | 0.86(0.03,21.69) | Moderate^Ⅱ^ | 0.53(0.31,0.89) | Moderate |
|  | Adalimumab vs Upadacitinib | 9.99(0.48,208.74) | High | 0.45(0.05,3.89) | High | 1.27(0.22,7.41) | Moderate^Ⅲ^ |
|  | Baricitinib vs Decernotinib | - | - | 0.44(0.03,5.63) | Moderate^Ⅱ^ | 0.44(0.03,5.63) | Very low^Ⅳ^ |
|  | Baricitinib vs Placebo | 0.64(0.15,2.76) | High | 8.08(0.04,1602.35) | High | 0.76(0.19,3.14) | Moderate^Ⅲ^ |
|  | Baricitinib vs Tofacitinib | - | - | 0.28(0.05,1.62) | Moderate^Ⅱ^ | 0.28(0.05,1.62) | Low^Ⅲ^ |
|  | Baricitinib vs Upadacitinib | - | - | 0.67(0.09,4.74) | High | 0.67(0.09,4.74) | Moderate^Ⅲ^ |
|  | Decernotinib vs Placebo | 1.73(0.21,14.37) | Moderate^Ⅰ^ | - | - | 1.73(0.21,14.37) | Low^Ⅲ^ |
|  | Decernotinib vs Tofacitinib | - | - | 0.63(0.05,7.57) | Moderate^Ⅱ^ | 0.63(0.05,7.57) | Very low^Ⅳ^ |
|  | Decernotinib vs Upadacitinib | - | - | 1.51(0.12,18.95) | Moderate^Ⅱ^ | 1.51(0.12,18.95) | Low^Ⅲ^ |
|  | Placebo vs Tofacitinib | 0.54(0.03,10) | Moderate^Ⅰ^ | 0.33(0.08,1.43) | Moderate^Ⅱ^ | 0.36(0.10,1.35) | Low^Ⅲ^ |
|  | Upadacitinib vs Placebo | 1.23（0.31，4.91） | High | 0.00(0.00,1152.24) | High | 1.14 (0.29，4.56) | Moderate^Ⅲ^ |
|  | Tofacitinib vs Upadacitinib | - | - | 2.41(0.39,14.86) | Moderate^Ⅱ^ | 2.41(0.39,14.86) | Low^Ⅲ^ |
| MACE | Abatacept vs Adalimumab | - | - | 0.25 (0.01,9.18) | High | 0.25 (0.01,9.18) | Low^Ⅳ^ |
|  | Abatacept vs Baricitinib | - | - | 0.32 (0.01,13.28) | High | 0.32 (0.01,13.28) | Low^Ⅳ^ |
|  | Abatacept vs Decernotinib | - | - | 0.44 (0.01,26.74) | Moderate^Ⅱ^ | 0.44 (0.01,26.74) | Very low^Ⅳ^ |
|  | Abatacept vs Placebo | - | - | 0.33 (0.01,10.19) | High | 0.33 (0.01,10.19) | Low^Ⅳ^ |
|  | Abatacept vs Tofacitinib | - | - | 0.19 (0.01,7.05) | Moderate^Ⅱ^ | 0.19 (0.01,7.05) | Very low^Ⅳ^ |
|  | Abatacept vs Upadacitinib | 0.33 (0.01,8.03) | High | - | - | 0.33 (0.01,8.03) | Low^Ⅳ^ |
|  | Adalimumab vs Baricitinib | 0.74(0.07,8.16) | High | 2.26(0.20,25.28) | High | 1.29 (0.23,7.07) | Moderate^Ⅲ^ |
|  | Adalimumab vs Decernotinib | - | - | 1.76 (0.13,23.40) | Moderate^Ⅱ^ | 1.76 (0.13,23.40) | Very low^Ⅳ^ |
|  | Adalimumab vs Placebo | 1.44(0.35,5.86) | High | 1.05(0.08,13.46) | High | 1.34 (0.39,4.58) | Moderate^Ⅲ^ |
|  | Adalimumab vs Tofacitinib | 0.75(0.51,1.10) | Moderate^Ⅰ^ | 1.45(0.05,40.25) | Moderate^Ⅱ^ | 0.76 (0.52,1.11) | Low^Ⅲ^ |
|  | Adalimumab vs Upadacitinib | 9.99(0.48,208.74) | High | 0.56(0.08,3.95) | High | 1.30 (0.25,6.73) | Moderate^Ⅲ^ |
|  | Baricitinib vs Decernotinib | - | - | 1.37 (0.09,20.75) | Moderate^Ⅱ^ | 1.37 (0.09,20.75) | Very low^Ⅳ^ |
|  | Baricitinib vs Placebo | 0.93(0.19,4.61) | High | 2.10(0.04,115.56) | High | 1.04 (0.23,4.60) | Moderate^Ⅲ^ |
|  | Baricitinib vs Tofacitinib | - | - | 0.59 (0.10,3.34) | Moderate^Ⅱ^ | 0.59 (0.10,3.34) | Low^Ⅲ^ |
|  | Baricitinib vs Upadacitinib | - | - | 1.01 (0.15,6.66) | High | 1.01 (0.15,6.66) | Moderate^Ⅲ^ |
|  | Decernotinib vs Placebo | 0.76 (0.08,7.35) | Moderate^Ⅰ^ | - | - | 0.76 (0.08,7.35) | Low^Ⅲ^ |
|  | Decernotinib vs Tofacitinib | - | - | 0.43 (0.03,5.80) | Moderate^Ⅱ^ | 0.43 (0.03,5.80) | Very low^Ⅳ^ |
|  | Decernotinib vs Upadacitinib | - | - | 0.74 (0.06,9.56) | Moderate^Ⅱ^ | 0.74 (0.06,9.56) | Very low^Ⅳ^ |
|  | Placebo vs Tofacitinib | 0.97(0.05,20) | Moderate^Ⅰ^ | 0.51(0.13,2.04) | Moderate^Ⅱ^ | 0.57 (0.16,2.02) | Low^Ⅲ^ |
|  | Upadacitinib vs Placebo | 1.05（0.32，3.43） | High | 0.00(0.00,960708.31) | High | 1.03 (0.32,3.35) | Moderate^Ⅲ^ |
|  | Tofacitinib vs Upadacitinib | - | - | 1.72 (0.32,9.16) | Moderate^Ⅱ^ | 1.72 (0.32,9.16) | Low^Ⅲ^ |
| Annotation：Ⅰ：Risk of bias exists； Ⅱ：Moderate quality (no low or very low quality) of evidence from indirect comparison；Ⅲ：Imprecision (wide confidence intervals including null effect values)；Ⅳ：Serious imprecision (very wide confidence intervals including null effect values)；"-" indicates none. This table is a supplement to Figure 3. | | | | | | | |

# Section 10. The results of sensitivity analyses

**Table S6. Results of sensitivity analyses**

| **By category** | | | | | | |
| --- | --- | --- | --- | --- | --- | --- |
|  | **Comparative intervention measures** | **Sensitivity analysis** | | | | |
|  |  | **Frequency** | **Bayesian** | **Risk of bias was rated as "some concern" for excluding low-cost interventions** | **Excluded studies that did not report all-cause mortality as an outcome measure** | **Excluded studies with follow-up periods less than 12 weeks** |
| **MACE** | Abatacept vs Adalimumab | 0.41 (0.02; 10.44) | 4.6e-07 (1.2e-14, 1.8264) | 0.23 (0.01; 7.78) | 0.42 (0.02; 10.45) | 0.22 (0.01; 7.44) |
|  | Abatacept vs JAK-i | 0.33 (0.01; 8.03) | 7.4e-08 (3.6e-19, 1.4869) | 0.33 (0.01; 8.03) | 0.33 (0.01; 8.03) | 0.33 (0.01; 8.03) |
|  | Abatacept vs Placebo | 0.38 (0.01; 10.20) | 8.5e-09 (2.1e-18, 3.4018) | 0.34 (0.01; 9.62) | 0.39 (0.01; 10.57) | 0.33 (0.01; 8.94) |
|  | Adalimumab vs JAK-i | 0.79 (0.54; 1.14) | 0.9054 (0.1646, 5.8131) | 1.41 (0.33; 6.05) | 0.78 (0.54; 1.14) | 1.46 (0.35; 6.05) |
|  | Adalimumab vs Placebo | 0.91 (0.41; 2.05) | 1.7326 (0.3249, 18.5619) | 1.48 (0.39; 5.60) | 0.93 (0.40; 2.19) | 1.46 (0.39; 5.49) |
|  | JAK-i vs Placebo | 1.16 (0.54; 2.50) | 1.8827 (0.5892, 10.5112) | 1.05 (0.41; 2.67) | 1.19 (0.53; 2.68) | 1.00 (0.44; 2.30) |
| **all-cause mortality** | Abatacept vs Adalimumab | 0.83 (0.07; 9.69) | 0.5079 (0.0045, 28.9383) | 0.23 (0.01; 7.97) | 0.83 (0.07; 9.69) | 0.22 (0.01; 7.53) |
|  | Abatacept vs JAK-i | 0.49 (0.04; 5.42) | 0.3914 (0.0052, 15.9534) | 0.33 (0.01; 8.03) | 0.49 (0.04; 5.42) | 0.33 (0.01; 8.03) |
|  | Abatacept vs Placebo | 0.69 (0.05; 8.66) | 0.7413 (0.009, 46.6823) | 0.35 (0.01; 10.05) | 0.69 (0.05; 8.66) | 0.33 (0.01; 9.15) |
|  | Adalimumab vs JAK-i | 0.59 (0.36; 0.98) | 0.7341 (0.1391, 5.1621) | 1.40 (0.32; 6.15) | 0.59 (0.36; 0.98) | 1.45 (0.34; 6.14) |
|  | Adalimumab vs Placebo | 0.83 (0.35; 1.98) | 1.4132 (0.2517, 14.7297) | 1.49 (0.39; 5.66) | 0.83 (0.35; 1.98) | 1.46 (0.39; 5.52) |
|  | JAK-i vs Placebo | 1.41 (0.63; 3.12) | 1.8744 (0.5611, 9.6962) | 1.07 (0.38; 2.98) | 1.41 (0.63; 3.12) | 1.01 (0.41; 2.47) |
| **By medication** | | | | | | |
| **MACE** | Abatacept vs Adalimumab | 0.25 (0.01; 9.18) | 1.2e-07 (1.4e-15, 1.85) | 0.23 (0.01; 8.61) | 0.26 (0.01; 9.98) | 0.23 (0.01; 8.61) |
|  | Abatacept vs Baricitinib | 0.32 (0.01; 13.28) | 2.3e-09 (1.2e-17, 4.65) | 0.32 (0.01; 13.01) | 0.33 (0.01; 14.54) | 0.32 (0.01; 13.01) |
|  | Abatacept vs Decernotinib | 0.44 (0.01; 26.74) | 8.3e-09 (4.2e-16, 0.0017) | - | 0.45 (0.01; 29.19) | 0.45 (0.01; 26.95) |
|  | Abatacept vs Placebo | 0.33 (0.01; 10.19) | 1.5e-12 (7.4e-25, 4.48) | 0.34 (0.01; 10.26) | 0.34 (0.01; 11.26) | 0.34 (0.01; 10.26) |
|  | Abatacept vs Tofacitinib | 0.19 (0.01; 7.05) | 6.1e-06 (3.1e-13, 1.26) | - | 0.19 (0.00; 7.67) | 0.33 (0.00; 31.84) |
|  | Abatacept vs Upadacitinib | 0.33 (0.01; 8.03) | 1.4e-07 (1.1e-15, 1.71) | 0.33 (0.01; 8.03) | 0.33 (0.01; 8.03) | 0.33 (0.01; 8.03) |
|  | Adalimumab vs Baricitinib | 1.29 (0.23; 7.07) | 2.22 (0.11, 54.31) | 1.39 (0.24; 7.99) | 1.29 (0.23; 7.09) | 1.39 (0.24; 7.99) |
|  | Adalimumab vs Decernotinib | 1.76 (0.13; 23.40) | 5.1e-08 (1.3e-16, 2.0826) | - | 1.77 (0.13; 23.46) | 1.96 (0.14; 27.51) |
|  | Adalimumab vs Placebo | 1.34 (0.39; 4.58) | 2.7581 (0.264, 41.7324) | 1.49 (0.39; 5.70) | 1.34 (0.39; 4.59) | 1.49 (0.39; 5.70) |
|  | Adalimumab vs Tofacitinib | 0.76 (0.52; 1.11) | 0.599 (0.0213, 8.7405) | - | 0.76 (0.52; 1.11) | 1.45 (0.05; 40.25) |
|  | Adalimumab vs Upadacitinib | 1.30 (0.25; 6.73) | 1.4442 (0.0688, 27.2059) | 1.44 (0.26; 8.01) | 1.27 (0.22; 7.49) | 1.44 (0.26; 8.01) |
|  | Baricitinib vs Decernotinib | 1.37 (0.09; 20.75) | 4.7e-07 (2.4e-13, 0.9487) | - | 1.37 (0.09; 20.76) | 1.41 (0.09; 21.44) |
|  | Baricitinib vs Placebo | 1.04 (0.23; 4.60) | 1.2435 (0.1038, 17.7702) | 1.07 (0.24; 4.77) | 1.04 (0.23; 4.60) | 1.07 (0.24; 4.77) |
|  | Baricitinib vs Tofacitinib | 0.59 (0.10; 3.34) | 0.2606 (0.0028, 10.8444) | - | 0.59 (0.10; 3.34) | 1.04 (0.04; 30.87) |
|  | Baricitinib vs Upadacitinib | 1.01 (0.15; 6.66) | 0.6544 (0.0212, 14.4402) | 1.03 (0.16; 6.83) | 0.99 (0.13; 7.39) | 1.03 (0.16; 6.83) |
|  | Decernotinib vs Placebo | 0.76 (0.08; 7.35) | 17616194988.4943 (1.6597, 3.23017047075561e+31) | - | 0.76 (0.08; 7.35) | 0.76 (0.08; 7.35) |
|  | Decernotinib vs Tofacitinib | 0.43 (0.03; 5.80) | 3216646613.5914 (0.2049, 6.04671713026262e+30) | - | 0.43 (0.03; 5.80) | 0.74 (0.02; 32.90) |
|  | Decernotinib vs Upadacitinib | 0.74 (0.06; 9.56) | 8550425008.6641 (0.7223, 1.65250546350443e+31) | - | 0.72 (0.05; 10.30) | 0.73 (0.06; 9.49) |
|  | Placebo vs Tofacitinib | 0.57 (0.16; 2.02) | 0.2144 (0.0031, 4.6821) | - | 0.57 (0.16; 2.02) | 0.97 (0.05; 20.40) |
|  | Placebo vs Upadacitinib | 0.97 (0.30; 3.17) | 0.5344 (0.0457, 3.4021) | 0.97 (0.30; 3.15) | 0.95 (0.24; 3.79) | 0.97 (0.30; 3.15) |
|  | Tofacitinib vs Upadacitinib | 1.72 (0.32; 9.16) | 2.5288 (0.0587, 179.5046) | - | 1.68 (0.28; 10.18) | 0.99 (0.04; 25.90) |
| **all-cause mortality** | Abatacept vs Adalimumab | 0.38 (0.02; 7.60) | 0.217 (0.001, 28.7305) | 0.23 (0.01; 9.37) | 0.38 (0.02; 7.60) | 0.23 (0.01; 9.37) |
|  | Abatacept vs Baricitinib | 0.73 (0.03; 16.28) | 0.6724 (0.0029, 123.5198) | 0.32 (0.01; 14.28) | 0.73 (0.03; 16.28) | 0.32 (0.01; 14.28) |
|  | Abatacept vs Decernotinib | 0.32 (0.01; 10.58) | 7.5e-09 (3.8e-17, 0.1503) | - | 0.32 (0.01; 10.58) | 0.46 (0.01; 29.50) |
|  | Abatacept vs Placebo | 0.56 (0.03; 8.96) | 0.5053 (0.0039, 53.5297) | 0.35 (0.01; 11.38) | 0.56 (0.03; 8.96) | 0.35 (0.01; 11.38) |
|  | Abatacept vs Tofacitinib | 0.20 (0.01; 4.13) | 0.0794 (2e-04, 14.3062) | - | 0.20 (0.01; 4.13) | 0.34 (0.00; 34.64) |
|  | Abatacept vs Upadacitinib | 0.49 (0.04; 5.42) | 0.3888 (0.0046, 19.4137) | 0.33 (0.01; 8.03) | 0.49 (0.04; 5.42) | 0.33 (0.01; 8.03) |
|  | Adalimumab vs Baricitinib | 1.90 (0.34; 10.51) | 3.098 (0.1747, 70.052) | 1.39 (0.24; 8.01) | 1.90 (0.34; 10.51) | 1.39 (0.24; 8.01) |
|  | Adalimumab vs Decernotinib | 0.84 (0.07; 9.78) | 1.3e-09 (4.3e-18, 0.4139) | - | 0.84 (0.07; 9.78) | 1.97 (0.14; 27.60) |
|  | Adalimumab vs Placebo | 1.45 (0.42; 5.04) | 2.3246 (0.2535, 29.6681) | 1.49 (0.39; 5.72) | 1.45 (0.42; 5.04) | 1.49 (0.39; 5.72) |
|  | Adalimumab vs Tofacitinib | 0.53 (0.31; 0.89) | 0.3947 (0.0154, 4.4516) | - | 0.53 (0.31; 0.89) | 1.45 (0.05; 40.38) |
|  | Adalimumab vs Upadacitinib | 1.27 (0.22; 7.41) | 1.7378 (0.0964, 38.1439) | 1.40 (0.22; 8.82) | 1.27 (0.22; 7.41) | 1.40 (0.22; 8.82) |
|  | Baricitinib vs Decernotinib | 0.44 (0.03; 5.63) | 3.3e-09 (9.5e-20, 0.1322) | - | 0.44 (0.03; 5.63) | 1.41 (0.09; 21.46) |
|  | Baricitinib vs Placebo | 0.76 (0.19; 3.14) | 0.7481 (0.0632, 9.5251) | 1.07 (0.24; 4.77) | 0.76 (0.19; 3.14) | 1.07 (0.24; 4.77) |
|  | Baricitinib vs Tofacitinib | 0.28 (0.05; 1.62) | 0.1221 (0.0014, 3.7745) | - | 0.28 (0.05; 1.62) | 1.04 (0.04; 30.90) |
|  | Baricitinib vs Upadacitinib | 0.67 (0.09; 4.74) | 0.5634 (0.019, 14.671) | 1.01 (0.13; 7.55) | 0.67 (0.09; 4.74) | 1.01 (0.13; 7.55) |
|  | Decernotinib vs Placebo | 1.73 (0.21; 14.37) | 36987694384.4476 (7.0068, 2.28280099881981e+35) | - | 1.73 (0.21; 14.37) | 0.76 (0.08; 7.35) |
|  | Decernotinib vs Tofacitinib | 0.63 (0.05; 7.57) | 5348525555.6793 (0.6687, 3.28468837210382e+34) | - | 0.63 (0.05; 7.57) | 0.74 (0.02; 32.90) |
|  | Decernotinib vs Upadacitinib | 1.51 (0.12; 18.95) | 27057092478.942 (4.4036, 1.73009022911242e+35) | - | 1.51 (0.12; 18.95) | 0.71 (0.05; 10.20) |
|  | Placebo vs Tofacitinib | 0.36 (0.10; 1.35) | 0.1686 (0.0029, 2.7094) | - | 0.36 (0.10; 1.35) | 0.97 (0.05; 20.40) |
|  | Placebo vs Upadacitinib | 0.87 (0.22; 3.48) | 0.7646 (0.0661, 6.6408) | 0.94 (0.24; 3.76) | 0.87 (0.22; 3.48) | 0.94 (0.24; 3.76) |
|  | Tofacitinib vs Upadacitinib | 2.41 (0.39; 14.86) | 4.5647 (0.1472, 374.9187) | - | 2.41 (0.39; 14.86) | 0.96 (0.03; 27.26) |

Annotation："-" indicates none
